# Supplementary material for: Shared and unique brain network features predict cognitive, personality, and mental health scores in the ABCD study
Source: Nat Commun. 2022 Apr 25;13:2217. doi: 10.1038/s41467-022-29766-8 (PMC9038754; doi:10.1038/s41467-022-29766-8)
Supplement: Supplementary file 1 — Supplementary Information [file 41467_2022_29766_MOESM1_ESM.pdf]

Supplementary Information for

Shared and unique brain network features predict cognitive, personality, and mental health scores in the ABCD study

Table of Contents

Supplementary methods and materials *2*

S1. MRI acquisition 2

S2. Behavioral data 2

S3. Multi-kernel ridge regression 4

S4. Predictive-feature matrices 6

Supplementary results *7*

Supplementary references *53*

Supplementary methods and materials

S1. MRI acquisition

For each participant, twenty minutes of resting-state fMRI data were acquired in four 5-minute runs. The task fMRI data consisted of three tasks (MID, N-back, SST) that were each acquired over two runs (for a total of six task fMRI runs). Each fMRI run was acquired in 2.4 mm isotropic resolution with a TR of 800 ms. The structural data consisted of one 1 mm isotropic scan for each participant. Full details of image acquisition can be found elsewhere (Casey *et al.* 2018).

S2. Behavioral data

We analyzed data from all available dimensional neurocognitive (Luciana *et al.* 2018) and mental health (Barch *et al.* 2018) assessments. For the neurocognitive assessments, we included the NIH Toolbox, Rey Auditory Verbal Learning Test, Little Man Task, and the matrix reasoning subscale from the Wechsler Intelligence Scale for Children-V, in order to measure different aspects of cognition. For the mental health assessments, we included the Achenbach Child Behavior Check List (CBCL), the mania scale from the Parent General Behavior Inventory, Pediatric Psychosis Questionnaire. For the personality measures, we included the Modified UPPS-P for Children and Behavioral Inhibition and Activation scales. See Supplementary Tables 1 & 2 for more details for each individual scale.

Supplementary Table 1. Behavioral measures used in this study. Behavioral measures are color coded by behavioral domain (red = cognition, blue = personality, green = mental health).

**Scale**	**Subscale/Measure**
NIH Toolbox (Hodes *et al.* 2013)	Flanker (attention)<br>List sorting working memory (working memory)<br>Dimensional change card sort (executive function)<br>Oral reading recognition (reading)<br>Pattern comparison processing speed (processing speed)<br>Picture sequence memory test (episodic memory)<br>Picture vocabulary test (vocabulary)<br>Cognition fluid composite (fluid cognition)<br>Crystallized composite (crystallized cognition)<br>Cognition total composite (total cognition)
Rey Auditory Verbal Learning Test (RAVLT) (Strauss *et al.* 2006)	Short delay recall<br>Long delay recall
Little Man Task (Acker and Acker 1982)	Accuracy (visuospatial accuracy)<br>Reaction time - correct responses (visuospatial reaction time)<br>Efficiency (visuospatial efficiency)
Wechsler Intelligence Scale for Children-V (WISC-V) (Wechsler 2014)	Matrix reasoning (fluid intelligence)
Modified UPPS-P for Children from PhenX (Lynam 2013)	Negative urgency<br>Positive urgency

	Lack of planning<br>Lack of perseverance<br>Sensation seeking
Behavioral Inhibition & Activation (Pagliaccio *et al.* 2016)	Behavioral inhibition sum<br>Reward responsiveness<br>Drive<br>Fun seeking
Achenbach Child Behavior Check List (Achenbach and Rescorla 2013)	Anxious/Depressed<br>Withdrawn/Depressed<br>Somatic complaints<br>Social problems<br>Thought problems<br>Attention problems<br>Rule-breaking behavior<br>Aggressive behavior
Parent General Behavior Inventory (Youngstrom *et al.* 2013)	Mania
Pediatric Psychosis Questionnaire - Brief Version (Loewy *et al.* 2012)	Total number of psychosis symptoms<br>Symptom severity score

Supplementary Table 2. Lookup table showing the original ABCD variable names with the corresponding descriptive labels used in the manuscript. More details of the behavioral measures can be found in the ABCD data dictionary. Behavioral measures are color coded by behavioral domain (red = cognition, blue = personality, green = mental health).

**Description**	**ABCD field**	**ABCD file**
Vocabulary	nihtbx_picvocab_uncorrected	abcd_tbss01.txt
Attention	nihtbx_flanker_uncorrected	abcd_tbss01.txt
Working memory	nihtbx_list_uncorrected	abcd_tbss01.txt
Executive function	nihtbx_cardsort_uncorrected	abcd_tbss01.txt
Processing speed	nihtbx_pattern_uncorrected	abcd_tbss01.txt
Episodic memory	nihtbx_picture_uncorrected	abcd_tbss01.txt
Reading	nihtbx_reading_uncorrected	abcd_tbss01.txt
Fluid cognition	nihtbx_fluidcomp_uncorrected	abcd_tbss01.txt
Crystallized cognition	nihtbx_cryst_uncorrected	abcd_tbss01.txt
Overall cognition	nihtbx_totalcomp_uncorrected	abcd_tbss01.txt
Short delay recall	pea_ravlt_sd_trial_vi_tc	abcd_ps01.txt
Long delay recall	pea_ravlt_ld_trial_vii_tc	abcd_ps01.txt

Fluid intelligence	pea_wiscv_trs	abcd_ps01.txt
Visuospatial accuracy	lmt_scr_perc_correct	lmtp201.txt
Visuospatial reaction time	lmt_scr_rt_correct	lmtp201.txt
Visuospatial efficiency	lmt_scr_efficiency	lmtp201.txt
Negative urgency	upps_y_ss_negative_urgency	abcd_mhy02.txt
Lack of planning	upps_y_ss_lack_of_planning	abcd_mhy02.txt
Sensation seeking	upps_y_ss_sensation_seeking	abcd_mhy02.txt
Positive urgency	upps_y_ss_positive_urgency	abcd_mhy02.txt
Lack perseverance	upps_y_lack_of_perseverance	abcd_mhy02.txt
Behavioral inhibition	bis_y_ss_bis_sum	abcd_mhy02.txt
Reward responsiveness	bis_y_ss_bas_rr	abcd_mhy02.txt
Drive	bis_y_ss_bas_drive	abcd_mhy02.txt
Fun seeking	bis_y_ss_bas_fs	abcd_mhy02.txt
Anxious depressed	cbcl_scr_syn_anxdep_r	abcd_cbcls01.txt
Withdrawn depressed	cbcl_scr_syn_withdep_r	abcd_cbcls01.txt
Somatic complaints	cbcl_scr_syn_somatic_r	abcd_cbcls01.txt
Social problems	cbcl_scr_syn_social_r	abcd_cbcls01.txt
Thought problems	cbcl_scr_syn_thought_r	abcd_cbcls01.txt
Attention problems	cbcl_scr_syn_attention_r	abcd_cbcls01.txt
Rule-breaking behavior	cbcl_scr_syn_rulebreak_r	abcd_cbcls01.txt
Aggressive behavior	cbcl_scr_syn_aggressive_r	abcd_cbcls01.txt
Total psychosis symptoms	pps_y_ss_number	abcd_mhy02.txt
Psychosis severity	pps_y_ss_severity_score	abcd_mhy02.txt
Mania	pgbi_p_ss_score	abcd_mhp02.txt

S3. Multi-kernel ridge regression

S3.1. Single-kernel ridge regression

For completeness, we provide a brief explanation of single-kernel ridge regression. The following section is adapted from our previous study (Kong et al., 2019). Suppose we have M training participants. Let y_i be the behavioral measure (e.g., fluid intelligence) and FC_i be the vectorized FC

(considering only lower triangular matrix) of the i-th training participant. Given $\{y_1, y_2, \cdots, y_M\}$ and $\{FC_1, FC_2, \cdots, FC_M\}$, the kernel regression model is written as:

$$y_i = \beta_0 + \sum_{j=1}^{M} \alpha_j K(FC_j, FC_i) \quad (1)$$

where β_0 is the bias term and $K(FC_j, FC_i)$ is the functional connectivity similarity between the i-th and j-th training participants. $K(FC_j, FC_i)$ is defined by the correlation between the vectorized FC of the two participants. The choice of correlation is motivated by previous fingerprinting and behavioral prediction studies (Finn *et al.* 2015, Li *et al.* 2019, He *et al.* 2020).

To estimate α and β_0 from the training set, let $\boldsymbol{y} = [y_1, y_2, \cdots, y_M]^T$, $\boldsymbol{\alpha} = [\alpha_1, \alpha_2, \cdots, \alpha_M]^T$ and $\mathbb{K}$ be the $M \times M$ kernel similarity matrix, whose (j, i)-th element is $K(FC_j, FC_i)$. Note that we can rewrite Eq. (1) as $\boldsymbol{y} = \mathbb{K}\boldsymbol{\alpha} + \beta_0$. We can then estimate $\boldsymbol{\alpha}$ and β_0 by minimizing the following l_2-regularized cost function:

$$(\boldsymbol{\alpha}, \beta_0) = \underset{(\boldsymbol{\alpha}, \beta_0)}{argmin} \frac{1}{2}(\boldsymbol{y} - \mathbb{K}\boldsymbol{\alpha} - \beta_0)^T(\boldsymbol{y} - \mathbb{K}\boldsymbol{\alpha} - \beta_0) + \frac{\lambda}{2}\boldsymbol{\alpha}^{\boldsymbol{T}}\mathbb{K}\boldsymbol{\alpha} \quad (2)$$

where λ controls the importance of the l_2-regularization and is estimated within the inner-loop cross-validation procedure. We emphasize that the test set was not used to estimate λ. Once $\boldsymbol{\alpha}$ and β_0 have been estimated from the training set, the predicted behavior of test participant t is given by:

$$\hat{y}_t = \beta_0 + \sum_{i=1}^{M} \alpha_i K(FC_i, FC_t) \quad (3)$$

S3.2. Multi-kernel ridge regression

Single-kernel ridge regression uses data from a single fMRI brain state for prediction. To extend to multiple fMRI brain states, we can construct one kernel similarity matrix for each fMRI brain state. Suppose we have M training participants and R fMRI brain states. Let y_i be the behavioral measure of the i-th training participant. Let FC_{ir} be the vectorized FC of the i-th training participant for the r-th fMRI brain state. The multi-kernel regression model can be written as

$$y_i = \beta_0 + \sum_{r=1}^{R}\sum_{j=1}^{M} \alpha_{jr} K\left(FC_{jr}, FC_{ir}\right) \quad (4)$$

where β_o is the bias term and $K(FC_{jr}, FC_{ir})$ is the functional connectivity similarity between the i-th and j-th training participants for the r-th brain state. Like before, $K(FC_{jr}, FC_{ir})$ is defined by the correlation between the vectorized FC of the two participants for the r-th brain state.

Let $\boldsymbol{y} = [y_1, y_2, \cdots, y_M]^T$ and $\boldsymbol{\alpha_r} = \left[\alpha_{1,r}, \alpha_{2,r}, \cdots, \alpha_{M,r}\right]^T$. Suppose $\mathbb{K}_{\boldsymbol{r}}$ is the $M \times M$ kernel similarity matrix for the r-th brain state, whose (j, i)-th element is $K(FC_{jr}, FC_{ir})$. We can estimate $\boldsymbol{\alpha_r}$ and β_0 by minimizing the following l_2-regularized cost function:

$$(\boldsymbol{\alpha_1}, \boldsymbol{\alpha_2}, \cdots, \boldsymbol{\alpha_R}, \beta_0) = \underset{\boldsymbol{\alpha}, \beta_0}{argmin} \frac{1}{2}\left(\boldsymbol{y} - \beta_0 - \sum_{i=1}^{R} \mathbb{K}_{\boldsymbol{r}}\boldsymbol{\alpha_r}\right)^T \left(\boldsymbol{y} - \beta_0 - \sum_{i=1}^{R} \mathbb{K}_{\boldsymbol{r}}\boldsymbol{\alpha_r}\right) + \frac{1}{2}\sum_{i=1}^{R} \lambda_r \boldsymbol{\alpha_r^T}\mathbb{K}\boldsymbol{\alpha} \quad (5)$$

where λ_r controls the importance of the l_2-regularization for the r-th kernel. Here, λ_r is estimated within the inner-loop cross-validation procedure using Gaussian-process optimization (Kawaguchi et al., 2015). We emphasize that the test set was not used to estimate λ_r. Once $\boldsymbol{\alpha_r}$ and β_0 have been estimated from the training set, the predicted behavior of test participant t is given by

$$y_t = \beta_0 + \sum_{r=1}^{R} \sum_{j=1}^{M} \alpha_{ir} K(FC_{ir}, FC_{tr}) \quad (6)$$

S3.3. Coefficient of determination (COD)

Suppose N is the number of test participants, y_t and $\hat{y}_t$ are the groundtruth and predicted behavior measure of the t-th test participant respectively, and $\bar{y}_{train}$ is the mean of the behavioral measure of all training participants. The coefficient of determination is defined as follows:

$$COD = 1 - \frac{\sum_{t=1}^{N} (\hat{y}_t - y_t)^2}{\sum_{t=1}^{N} (\bar{y}_{train} - y_t)^2} \quad (7)$$

Thus, a larger COD indicates more accurate prediction. A negative value implies that we are better off using the mean behavior of the training participants to predict the behavior of the test participant instead of using the FC data.

S4. Predictive-feature matrices

To interpret which brain edges were important for the multi-kernel FC model, we utilized an elegant approach (Haufe *et al.* 2014) to invert the prediction model. Failure to invert the model leads to uninterpretable results (Haufe *et al.* 2014). Let us consider the functional connectivity between brain regions a and b. We would like to compute the predictive-feature value of the functional connection p_{ab} for the multi-kernel FC model. A positive value (or negative) predictive-feature value for an edge indicates that higher FC between brain regions a and b is associated with predicting greater (or lower) behavioral values.

The FC of each participant was normalized to achieve zero mean and unit norm across all edges of the participant. This normalization arises from our choice of the correlation metric in the kernel ridge regression model. Not performing the normalization results in highly similar predictive network feature matrices (not shown). Let FC_{ab} be the normalized functional connectivity strength between brain regions a and b for all training participants, i.e., FC_{ab} is an $N \times 1$ vector where N is the number of training participants. Let $\hat{y}$ be the prediction of the training participants' behavioral measure based on the estimated kernel regression model. Therefore, $\hat{y}$ is an $N \times 1$ vector where N is the number of training participants. According to Haufe and colleagues (2014), $p_{ab} = covariance(FC_{ab}, \hat{y})$.

However, because we would like to compare across different behavioral measures, the scale of $\hat{y}$ is very different across behavioral measures. Thus, we computed $p_{ab} = covariance(FC_{ab}, \hat{y})/variance(\hat{y})$, which does not change the relative predictive-feature values among edges, but allows for comparisons between behavioral measures. We note that the above formula is applied to the training set, because we want to interpret the trained model. However, recall that we performed leave-3-site-clusters-out nested cross-validation for each behavioral measure with 120 replications. Thus we computed the predictive-feature values for each replication and averaged across the 120 replications.

Supplementary results

Supplementary Table 3. Demographic information for included and excluded participants in ABCD 2.0.1

	**Included**	**Excluded**
**N**	1858	10017
**Age in months (mean (s.d.))**	120.34 (7.43)	118.68 (7.44)
**Female (%)**	1025 (55.17)	4656 (46.48)
**Race/ethnicity (%)**		
**Asian**	55 (2.96)	197 (1.97)
**Black**	143 (7.70)	1636 (16.33)
**Hispanic**	324 (17.44)	2083 (20.79)
**White**	1145 (61.63)	5029 (50.20)
**Other**	187 (10.06)	1058 (10.56)
**Unknown**	4 (0.21)	14 (0.14)
**Household income (%)**		
**< 50 000**	360 (19.38)	2862 (28.57)
**≥ 50 000 & < 100 000**	517 (27.82)	2553 (25.49)
**≥ 100 000**	873 (46.99)	3692 (36.86)
**Unknown**	108 (5.81)	910 (9.08)

Supplementary Table 4. Behavioral distribution of included and excluded participants in ABCD 2.0.1. 32 of the 36 behavioral measures (**bolded**) were statistically different between included and excluded participants after multiple comparisons correction with FDR q < 0.05.

	**Included (1858)**	**Excluded (10017)**
**Vocabulary**	86.45(7.75)	84.08(8.13)
**Attention**	95.62(7.98)	93.7(9.31)
**Working memory**	99.28(10.86)	96.14(12.24)
**Executive function**	94.84(8.09)	92.08(9.69)
**Processing speed**	90.80(13.98)	87.54(14.64)
**Episodic memory**	105.35(11.84)	102.33(12.06)
**Reading**	92.52(6.53)	90.54(6.93)
**Fluid cognition**	94.84(9.40)	90.92(10.77)
**Crystallized cognition**	88.33(6.61)	85.99(7.09)
**Overall cognition**	89.33(8.02)	85.63(9.22)
**Short delay recall**	10.22(2.83)	9.55(3.08)
**Long delay recall**	9.74(2.99)	9.06(3.23)
**Fluid intelligence**	18.81(3.56)	17.73(3.86)
**Visuospatial accuracy**	0.63(0.17)	0.58(0.17)
**Visuospatial reaction time**	2725.04(447.01)	2649.83(474.02)
**Visuospatial efficiency**	2.4e-4(7.3e-5)	2.3e-4(6.9e-5)
**Negative urgency**	8.31(2.51)	8.52(2.67)
**Lack of planning**	7.57(2.18)	7.77(2.41)
Sensation seeking	9.74(2.65)	9.77(2.69)
**Positive urgency**	7.63(2.77)	8.06(2.99)
**Lack perseverance**	6.81(2.12)	7.09(2.27)
Behavioral inhibition	9.46(3.62)	9.52(3.78)
**Reward responsiveness**	10.73(2.89)	11.05(2.92)
**Drive**	3.69(2.81)	4.22(3.10)
**Fun seeking**	5.57(2.48)	5.73(2.67)
Anxious depressed	2.51(2.93)	2.52(3.09)

**Withdrawn depressed**	0.95(1.60)	1.05(1.73)
Somatic complaints	1.51(1.92)	1.49(1.96)
**Social problems**	1.36(1.97)	1.67(2.33)
**Thought problems**	1.49(1.98)	1.64(2.23)
**Attention problems**	2.39(3.11)	3.09(3.55)
**Rule-breaking behavior**	0.94(1.51)	1.24(1.91)
**Aggressive behavior**	2.82(3.74)	3.35(4.45)
**Total psychosis symptoms**	2.08(3.18)	2.74(3.62)
**Psychosis severity**	4.83(8.91)	6.60(10.87)
**Mania**	0.99(2.22)	1.36(2.86)

Supplementary Table 5. Correlation between behavioral scores and mean framewise displacement (FD) across the included participants (n=1858).

	FD (Rest)	FD (MID)	FD (SST)	FD (N-back)
Vocabulary	-0.107	-0.010	-0.097	-0.100
Attention	-0.045	-0.056	-0.046	-0.053
Working memory	-0.080	-0.051	-0.025	-0.071
Executive function	-0.110	-0.069	-0.068	-0.075
Processing speed	-0.137	-0.097	-0.084	-0.084
Episodic memory	-0.089	-0.052	-0.036	-0.105
Reading	-0.103	-0.071	-0.089	-0.079
Fluid cognition	-0.151	-0.105	-0.082	-0.126
Crystallized cognition	-0.120	-0.096	-0.105	-0.101
Overall cognition	-0.164	-0.120	-0.109	-0.136
Short delay recall	-0.042	-0.045	-0.064	-0.066
Long delay recall	-0.055	-0.049	-0.060	-0.079
Fluid intelligence	-0.100	-0.077	-0.086	-0.093
Visuospatial accuracy	-0.121	-0.087	-0.067	-0.090
Visuospatial reaction time	0.025	0	0.016	0.005
Visuospatial efficiency	-0.120	-0.081	-0.068	-0.085
Negative urgency	0.046	0.043	0.028	0.036
Lack of planning	0.027	0.028	0.024	0.033
Sensation seeking	-0.009	0.002	-0.039	0.004
Positive urgency	0.104	0.101	0.069	0.091
Lack perseverance	-0.004	0.007	0.012	0.014
Behavioral inhibition	0.011	-0.008	0	0.047
Reward responsiveness	0.039	0.030	0.018	0.049
Drive	0.064	0.072	0.041	0.085
Fun seeking	0.087	0.081	0.046	0.063
Anxious depressed	-0.013	-0.016	-0.020	-0.007
Withdrawn depressed	-0.017	-0.015	-0.026	-0.005

Somatic complaints	-0.017	-0.040	-0.021	-0.005
Social problems	0.013	0.018	0.012	0.047
Thought problems	-0.004	0.007	-0.011	0.016
Attention problems	0.032	0.006	-0.011	0.042
Rule-breaking behavior	0.032	0.008	0.016	0.041
Aggressive behavior	0.010	-0.010	-0.003	0.017
Total psychosis symptoms	0.068	0.079	0.077	0.095
Psychosis severity	0.065	0.063	0.065	0.081
Mania	0.018	-0.006	-0.012	0.009

Supplementary Table 6. Correlation between behavioral scores and mean DVARS across the included participants (n=1858).

	**DVARS (Rest)**	**DVARS (MID)**	**DVARS (SST)**	**DVARS (N-back)**
Vocabulary	-0.077	-0.029	-0.040	-0.063
Attention	-0.0060	0.0010	-0.017	-0.025
Working memory	-0.023	0.0080	0.014	-0.025
Executive function	-0.029	0.019	-0.0070	-0.011
Processing speed	-0.044	0.044	0.014	0.013
Episodic memory	-0.064	-0.022	-0.0080	-0.067
Reading	-0.067	0.0050	-0.029	-0.042
Fluid cognition	-0.057	0.018	0.0020	-0.035
Crystallized cognition	-0.083	-0.016	-0.041	-0.061
Overall cognition	-0.081	0.0040	-0.020	-0.054
Short delay recall	-0.025	-0.0060	-0.028	-0.043
Long delay recall	-0.040	-0.0060	-0.029	-0.052
Fluid intelligence	-0.026	0.011	-0.012	-0.035
Visuospatial accuracy	-0.053	-0.0010	-0.013	-0.033
Visuospatial reaction time	0.0020	-0.0020	0.0080	-0.0090
Visuospatial efficiency	-0.048	-0.0040	-0.016	-0.026
Negative urgency	0.063	0.040	0.050	0.039
Lack of planning	0.0070	-0.012	-0.0020	-0.022
Sensation seeking	-0.018	-0.033	-0.050	-0.043
Positive urgency	0.089	0.079	0.072	0.079
Lack perseverance	0.067	0.059	0.054	0.059
Behavioral inhibition	0.037	0.049	0.030	0.061
Reward responsiveness	0.035	0.036	0.0030	0.023
Drive	0.036	0.039	0.017	0.053
Fun seeking	0.060	0.039	0.011	0.019
Anxious depressed	0.022	0.025	0.026	0.024

Withdrawn depressed	0.041	0.033	0.014	0.043
Somatic complaints	-0.0090	-0.020	-0.016	0.0030
Social problems	0.050	0.028	0.028	0.053
Thought problems	0.053	0.033	0.015	0.031
Attention problems	0.106	0.055	0.049	0.079
Rule-breaking behavior	0.050	0.011	0.012	0.028
Aggressive behavior	0.055	0.016	0.033	0.024
Total psychosis symptoms	0.041	0.0050	0.014	0.036
Psychosis severity	0.034	0.0080	0.012	0.037
Mania	0.053	0.021	0.0070	0.024

Supplementary Table 7. Correlation between behavioral scores and mean boundary-based registration (BBR) cost across the included participants (n=1858). A larger BBR value indicates worse alignment between the anatomical and functional images.

	BBR (REST)	BBR (MID)	BBR (SST)	BBR (N-back)
Vocabulary	0.033	0.003	-0.003	0.009
Attention	-0.002	-0.014	-0.003	0.017
Working memory	0.015	-0.014	0.006	-0.002
Executive function	-0.004	-0.014	0	0.009
Processing speed	-0.014	-0.022	-0.017	-0.012
Episodic memory	-0.021	-0.033	-0.031	-0.028
Reading	-0.007	-0.041	-0.019	-0.018
Fluid cognition	-0.009	-0.031	-0.017	-0.008
Crystallized cognition	0.016	-0.021	-0.012	-0.004
Overall cognition	0	-0.033	-0.018	-0.010
Short delay recall	0.006	-0.003	0.001	-0.016
Long delay recall	0.019	0.015	0.010	0.001
Fluid intelligence	-0.007	-0.028	-0.025	-0.013
Visuospatial accuracy	-0.047	-0.043	-0.045	-0.046
Visuospatial reaction time	0.002	0.010	0.002	0.009
Visuospatial efficiency	-0.042	-0.044	-0.038	-0.048
Negative urgency	-0.019	-0.010	-0.029	-0.024
Lack of planning	-0.014	-0.007	-0.010	0.004
Sensation seeking	-0.034	-0.040	-0.030	-0.035
Positive urgency	-0.039	-0.002	-0.030	-0.029
Lack perseverance	0.024	0.020	0.001	0.011
Behavioral inhibition	-0.051	-0.062	-0.053	-0.051
Reward responsiveness	-0.044	-0.043	-0.037	-0.023
Drive	-0.026	-0.032	-0.031	-0.023
Fun seeking	-0.021	-0.005	-0.005	-0.012
Anxious depressed	0.015	0.002	0.003	0

Withdrawn depressed	-0.024	-0.015	-0.028	-0.026
Somatic complaints	-0.003	-0.002	0.023	-0.003
Social problems	-0.019	-0.011	-0.017	-0.010
Thought problems	-0.026	-0.010	-0.026	-0.023
Attention problems	0.008	0.020	-0.007	0.007
Rule-breaking behavior	0.002	0.022	-0.012	0.024
Aggressive behavior	0.016	0.017	-0.011	0.020
Total psychosis symptoms	-0.031	-0.007	-0.028	-0.024
Psychosis severity	-0.021	-0.009	-0.029	-0.021
Mania	-0.03	0.003	-0.026	-0.022

Supplementary Table 8. Distribution of the included sample (n=1858) by site and scanner

ABCD Site	Make	Model	N	Site-cluster
16	Siemens	Prisma	292	A
13	GE	Discovery MR750	161	B
4	GE	Discovery MR750	145	C
22	GE	Discovery MR750	12	C
14	Siemens	Prisma/Prisma fit	135	D
15	Siemens	Prisma fit	27	D
6	Siemens	Prisma fit	131	E
9	Siemens	Prisma fit	52	E
10	GE	Discovery MR750	127	F
11	Siemens	Prisma	52	F
3	Siemens	Prisma	120	G
5	Siemens	Prisma fit	56	G
2	Siemens	Prisma fit	110	H
7	Siemens	Prisma fit	55	H
8	GE	Discovery MR750	63	I
20	Siemens	Prisma/Prisma fit	92	I
12	Siemens	Prisma fit	79	J
18	GE	Discovery MR750	73	J
21	Siemens	Prisma fit/Prisma	76	J

Supplementary Table 9A. Distributions of age, sex, and behavioral measures of included and excluded participants after matching. There is no significant difference in terms of age, sex and behavioral measures between included and excluded participants after multiple comparisons correction with FDR q < 0.05.

Behavior being matched	N matched	Included			Excluded		
		Age	Female	Behavior	Age	Female	Behavior
Vocabulary	924	119.33(7.44)	50.00	85.10(7.91)	118.90(7.46)	47.61	84.40(8.13)
Attention	910	119.15(7.47)	50.44	94.52(8.48)	118.92(7.46)	47.54	93.96(9.19)
Working memory	847	119.30(7.64)	50.65	97.39(11.70)	118.91(7.45)	47.55	96.59(12.11)
Executive function	563	119.45(7.61)	50.09	93.28(8.88)	118.91(7.46)	47.71	92.48(9.54)
Processing speed	998	119.34(7.46)	49.90	89.15(13.89)	118.90(7.46)	47.55	87.95(14.64)
Episodic memory	1002	119.36(7.51)	50.80	103.66(11.74)	118.90(7.46)	47.60	102.74(12.10)
Reading	1014	119.41(7.58)	50.30	91.54(6.73)	118.89(7.45)	47.60	90.79(6.92)
Fluid cognition	687	119.27(7.56)	50.80	92.40(10.57)	118.92(7.46)	47.61	91.50(10.66)
Crystallized cognition	748	119.23(7.66)	49.33	86.97(7.04)	118.92(7.45)	47.60	86.32(7.06)
Overall cognition	856	119.29(7.56)	50.70	87.17(8.30)	118.92(7.45)	47.56	86.15(9.20)
Short delay recall	1008	119.30(7.53)	50.99	9.76(2.83)	118.92(7.46)	47.57	9.65(3.07)
Long delay recall	924	119.40(7.46)	50.00	9.23(3.19)	118.92(7.46)	47.49	9.17(3.20)
Fluid intelligence	1073	119.40(7.50)	51.44	18.20(3.70)	118.90(7.46)	47.50	17.88(3.85)
Visuospatial accuracy	924	119.32(7.51)	50.32	0.60(0.17)	118.90(7.46)	47.50	0.59(0.17)
Visuospatial reaction time	836	119.38(7.42)	50.24	2685.85(477.99)	118.90(7.47)	47.72	2660.13(470.00)
Visuospatial efficiency	1005	119.25(7.42)	50.25	2.3e-4(7.4e-4)	118.91(7.47)	47.41	2.3e-4(6.9e-4)
Negative urgency	926	119.49(7.50)	50.11	8.47(2.56)	118.90(7.46)	47.58	8.49(2.65)
Lack of planning	847	119.40(7.46)	49.82	7.62(2.23)	118.91(7.46)	47.57	7.75(2.39)
Sensation seeking	769	119.44(7.45)	49.93	9.73(2.73)	118.91(7.46)	47.50	9.77(2.68)
Positive urgency	1004	119.40(7.59)	50.50	7.87(2.82)	118.91(7.45)	47.46	8.00(2.97)
Lack perseverance	1011	119.35(7.44)	50.45	7.05(2.21)	118.91(7.46)	47.49	7.04(2.26)
Behavioral inhibition	1002	119.42(7.59)	50.50	9.47(3.63)	118.91(7.45)	47.42	9.51(3.77)
Reward responsiveness	850	119.37(7.48)	50.35	10.95(2.90)	118.92(7.46)	47.48	11.01(2.92)
Drive	995	119.43(7.59)	51.06	3.96(2.93)	118.90(7.45)	47.47	4.16(3.07)
Fun seeking	844	119.43(7.58)	50.12	5.61(2.50)	118.91(7.45)	47.46	5.71(2.65)
Anxious depressed	996	119.33(7.59)	50.20	2.61(3.08)	118.91(7.45)	47.41	2.51(3.06)
Withdrawn depressed	928	119.26(7.54)	50.22	1.12(1.88)	118.92(7.46)	47.49	1.03(1.69)
Somatic complaints	997	119.38(7.56)	50.35	1.54(2.05)	118.90(7.45)	47.45	1.49(1.95)
Social problems	911	119.33(7.39)	49.73	1.59(2.27)	118.91(7.47)	47.44	1.63(2.28)
Thought problems	992	119.33(7.57)	50.30	1.60(2.21)	118.91(7.45)	47.45	1.62(2.19)
Attention problems	921	119.27(7.49)	50.05	2.92(3.58)	118.91(7.46)	47.42	2.98(3.49)
Rule-breaking behavior	924	119.38(7.66)	49.68	1.09(1.62)	118.90(7.44)	47.56	1.20(1.88)
Aggressive behavior	918	119.35(7.53)	50.11	3.17(4.12)	118.91(7.46)	47.47	3.27(4.37)
Total psychosis symptoms	996	119.33(7.48)	50.80	2.43(3.51)	118.91(7.46)	47.49	2.65(3.56)
Psychosis severity	1004	119.35(7.50)	50.20	5.81(9.73)	118.91(7.46)	47.51	6.37(10.69)
Mania	997	119.38(7.51)	50.25	1.24(2.65)	118.90(7.46)	47.54	1.31(2.79)

Supplementary Table 9B. Continuation of Supplementary Table 9. Distribution of racial composition of included and excluded participants after matching. There is no significant difference in terms of racial composition between included and excluded participants after multiple comparisons correction with FDR q < 0.05.

	Include					Excluded				
	Asian	Black	Hispanic	White	Other	Asian	Black	Hispanic	White	Other
Vocabulary	3.25	12.34	20.45	52.71	11.26	2.06	15.11	20.28	52.12	10.39
Attention	3.08	12.31	20.22	53.08	11.32	2.08	15.11	20.30	52.03	10.47
Working memory	3.19	13.11	20.54	52.07	11.10	2.08	15.02	20.26	52.12	10.43
Executive function	3.91	14.56	20.25	48.85	12.43	2.07	14.92	20.31	52.30	10.34
Processing speed	3.01	11.62	20.14	54.11	11.12	2.07	15.19	20.31	51.96	10.47
Episodic memory	3.19	11.98	20.16	53.79	10.88	2.06	15.17	20.30	52.02	10.47
Reading	3.35	11.83	19.72	54.04	11.05	2.04	15.19	20.36	52.01	10.39
Fluid cognition	3.78	13.10	21.11	49.93	12.08	2.06	14.97	20.21	52.42	10.35
Crystallized cognition	3.48	13.37	20.45	51.34	11.36	2.06	15.01	20.31	52.05	10.35
Overall cognition	3.50	12.27	20.56	51.87	11.80	2.05	15.07	20.24	52.37	10.36
Short delay recall	3.08	12.00	20.34	53.77	10.81	2.07	15.27	20.13	52.11	10.43
Long delay recall	3.03	20.78	53.03	12.34	10.82	2.09	20.15	52.12	15.34	10.43
Fluid intelligence	3.08	11.56	20.32	53.87	11.18	2.04	15.39	20.18	52.20	10.29
Visuospatial accuracy	3.03	12.45	20.56	52.81	11.15	2.03	15.25	20.26	51.86	10.45
Visuospatial reaction time	3.23	13.04	20.81	51.91	11.00	2.02	15.18	20.25	51.93	10.42
Visuospatial efficiency	2.89	12.54	20.50	53.13	10.95	2.04	15.26	20.27	51.65	10.41
Negative urgency	3.35	12.20	20.52	52.70	11.23	2.03	15.23	20.24	51.98	10.49
Lack of planning	3.19	12.75	20.66	51.59	11.81	2.05	15.16	20.23	52.02	10.45
Sensation seeking	3.25	12.87	21.46	50.98	11.44	2.05	15.14	20.18	51.79	10.52
Positive urgency	3.09	11.85	20.12	53.78	11.16	2.04	15.28	20.28	51.69	10.47
Lack perseverance	2.97	11.97	20.67	53.21	11.18	2.05	15.27	20.23	51.93	10.46
Behavioral inhibition	3.09	12.08	20.06	53.69	11.08	2.03	15.27	20.28	51.91	10.47
Reward responsiveness	3.18	12.71	20.35	52.00	11.76	2.04	15.18	20.25	51.80	10.50
Drive	3.12	12.16	19.60	53.57	11.56	2.03	15.26	20.32	51.84	10.50
Fun seeking	3.32	12.91	20.62	51.90	11.26	2.03	15.16	20.23	51.85	10.51
Anxious depressed	2.71	12.25	20.58	53.51	10.94	2.06	15.27	20.26	51.64	10.44
Withdrawn depressed	3.02	12.50	20.80	52.37	11.31	2.04	15.23	20.25	51.73	10.43
Somatic complaints	2.61	12.14	20.46	53.56	11.23	2.07	15.28	20.27	51.78	10.43
Social problems	2.85	12.18	20.64	52.91	11.42	2.06	15.25	20.26	51.82	10.47
Thought problems	2.62	12.00	20.06	54.13	11.19	2.07	15.29	20.31	51.56	10.45
Attention problems	2.82	12.38	20.41	52.99	11.40	2.06	15.24	20.28	51.72	10.49
Rule-breaking behavior	3.03	12.45	20.67	52.71	11.15	2.04	15.23	20.26	51.73	10.48
Aggressive behavior	2.83	12.42	20.70	52.72	11.33	2.06	15.23	20.25	51.80	10.44
Total psychosis symptoms	2.71	12.15	19.98	54.02	11.14	2.06	15.26	20.32	51.92	10.34
Psychosis severity	2.89	12.35	20.52	53.09	11.16	2.05	15.24	20.27	51.82	10.40
Mania	2.61	12.04	20.66	53.46	11.23	2.08	15.27	20.27	51.83	10.45

Supplementary Table 9C. Continuation of Supplementary Table 9. Distribution of family income of included and excluded participants after matching. There is no significant difference in terms of family income between included and excluded participants after multiple comparisons correction with FDR $q < 0.05$.

Behaviors being matched	Included				Excluded			
	< 50K	>= 50K & < 100K	>= 100K	Unknown	< 50K	>= 50K & < 100K	>= 100K	Unknown
Vocabulary	26.41	26.62	38.74	8.23	27.10	25.85	38.46	8.59
Attention	25.16	27.14	39.45	8.24	27.20	25.80	38.42	8.58

Working memory	25.97	26.33	39.08	8.62	27.13	25.89	38.48	8.50
Executive function	26.64	25.75	37.30	10.3	27.09	25.91	38.54	8.47
Processing speed	25.05	27.15	39.58	8.22	27.24	25.81	38.37	8.58
Episodic memory	25.75	26.75	39.52	7.98	27.16	25.83	38.40	8.61
Reading	25.74	26.92	38.95	8.38	27.17	25.83	38.44	8.56
Fluid cognition	26.20	26.49	37.55	9.75	27.07	25.89	38.63	8.42
Crystallized cognition	26.87	26.07	38.24	8.82	27.07	25.91	38.50	8.52
Overall cognition	25.93	27.22	37.73	9.11	27.10	25.83	38.61	8.45
Short delay recall	25.79	26.79	38.99	8.43	27.22	25.78	38.49	8.51
Long delay recall	26.19	26.30	38.74	8.77	27.16	25.83	38.54	8.46
Fluid intelligence	25.07	27.12	40.26	7.55	27.34	25.75	38.28	8.62
Visuospatial accuracy	25.97	26.19	39.61	8.23	26.99	25.89	38.51	8.61
Visuospatial reaction time	26.20	26.56	38.64	8.61	26.98	25.86	38.59	8.57
Visuospatial efficiency	25.37	26.57	39.60	8.46	27.07	25.85	38.49	8.58
Negative urgency	26.13	26.57	38.66	8.64	27.20	25.85	38.43	8.52
Lack of planning	26.09	25.97	38.72	9.21	27.20	25.90	38.43	8.47
Sensation seeking	26.66	26.27	37.71	9.36	27.15	25.88	38.50	8.47
Positive urgency	26.29	26.69	39.14	7.87	27.19	25.84	38.38	8.59
Lack perseverance	25.62	27.20	39.07	8.11	27.26	25.79	38.39	8.57
Behavioral inhibition	25.55	26.75	39.32	8.38	27.26	25.83	38.36	8.55
Reward responsiveness	26.59	26.59	38.12	8.71	27.16	25.86	38.47	8.51
Drive	26.13	26.33	39.40	8.14	27.21	25.87	38.36	8.56
Fun seeking	26.42	25.95	38.51	9.12	27.17	25.91	38.44	8.48
Anxious depressed	26.00	26.71	38.76	8.53	27.26	25.81	38.40	8.53
Withdrawn depressed	26.19	26.40	38.58	8.84	27.23	25.84	38.42	8.51
Somatic complaints	26.18	26.48	38.72	8.63	27.24	25.83	38.41	8.52
Social problems	26.02	26.45	38.86	8.67	27.24	25.84	38.40	8.52
Thought problems	26.21	26.31	39.21	8.27	27.24	25.85	38.36	8.56
Attention problems	26.60	26.17	38.98	8.25	27.20	25.86	38.39	8.56
Rule-breaking behavior	26.19	27.06	38.74	8.01	27.23	25.78	38.41	8.58
Aggressive behavior	25.93	26.36	39.32	8.39	27.25	25.84	38.36	8.54
Total psychosis symptoms	25.50	27.01	39.26	8.23	27.30	25.79	38.36	8.54
Psychosis severity	26.20	26.49	39.24	8.07	27.24	25.83	38.37	8.56
Mania	25.98	26.48	39.12	8.43	27.28	25.82	38.37	8.54

Supplementary Table 9D. Continuation of Supplementary Table 9. Uncorrected p values of differences in characteristics between included and excluded participants after matching. There is no significant difference after multiple comparisons correction with FDR q < 0.05.

	**Age**	**Sex**	**Income**	**Race**	**Behavior**
Vocabulary	0.10	0.18	0.92	0.03	0.01
Attention	0.38	0.11	0.53	0.06	0.07
Working memory	0.15	0.10	0.91	0.15	0.06
Executive function	0.10	0.29	0.50	0.02	0.05

Processing speed	0.07	0.19	0.43	0.01	0.01
Episodic memory	0.06	0.06	0.64	0.01	0.02
Reading	0.04	0.11	0.75	0.004	0.001
Fluid cognition	0.24	0.12	0.61	0.01	0.03
Crystallized cognition	0.28	0.41	0.99	0.08	0.01
Overall cognition	0.16	0.08	0.67	0.01	0.008
Short delay recall	0.12	0.04	0.77	0.02	0.24
Long delay recall	0.06	0.17	0.93	0.07	0.59
Fluid intelligence	0.03	0.01	0.18	0.004	0.01
Visuospatial accuracy	0.11	0.13	0.86	0.06	0.08
Visuospatial reaction time	0.08	0.16	0.96	0.09	0.13
Visuospatial efficiency	0.16	0.12	0.69	0.09	0.05
Negative urgency	0.02	0.16	0.91	0.01	0.78
Lack of planning	0.07	0.24	0.83	0.05	0.14
Sensation seeking	0.06	0.24	0.83	0.07	0.65
Positive urgency	0.04	0.08	0.75	0.01	0.16
Lack perseverance	0.08	0.09	0.58	0.02	0.94
Behavioral inhibition	0.04	0.08	0.67	0.02	0.73
Reward responsiveness	0.09	0.13	0.96	0.05	0.60
Drive	0.03	0.04	0.82	0.01	0.05
Fun seeking	0.05	0.18	0.91	0.05	0.26
Anxious depressed	0.08	0.12	0.84	0.09	0.29
Withdrawn depressed	0.18	0.14	0.90	0.06	0.13
Somatic complaints	0.05	0.10	0.91	0.08	0.44
Social problems	0.11	0.24	0.88	0.07	0.62
Thought problems	0.09	0.11	0.88	0.06	0.76
Attention problems	0.16	0.17	0.96	0.10	0.59
Rule-breaking behavior	0.06	0.25	0.75	0.06	0.09
Aggressive behavior	0.09	0.16	0.83	0.10	0.51

Total psychosis symptoms	0.08	0.06	0.59	0.06	0.06
Psychosis severity	0.07	0.13	0.81	0.06	0.11
Mania	0.05	0.12	0.83	0.07	0.49

Supplementary Table 10. List of statistical tests performed in the current study. There were four sets of FDR correction (q < 0.05) corresponding to four different sets of analyses.

**First set of statistical corrections (total of 105 statistical tests)**	
Figure 2	Compare all prediction models against chance-level prediction (5 models * 3 behavioral domains = 15 tests)<br>Compare prediction of resting-FC against each task-FC (3 tasks * 3 behavioral domains = 9 tests)<br>Compare multi-kernel FC against the best single kernel (3 behavioral domains = 3 tests)
Figure 3	Compare predictions against chance-level prediction (36 tests)
Supplementary Figure 2	Compare predictions against chance-level prediction (36 tests)
Supplementary Figure 18	Compare multi-kernel FC against mean-FC (3 behavioral domains = 3 tests)<br>Compare mean-FC model against the best single-kernel model (3 behavioral domains = 3 tests)
**Second set of statistical corrections (total of 4149 statistical tests)**	
Figure 5	Compare within- and between- domain network overlap against chance-level overlap (6 tests)<br>Compare within-domain overlap and between-domain overlap (6 tests)<br>Compare within brain state network overlap against chance-level overlap (3 tests)
Figure 7a	Compare model-transfer accuracies against chance-level prediction (9 tests)<br>Compare within-domain model-transfer against between-domain model transfer (6 tests)
Figure 7c	Compare feature-transfer accuracies against chance-level prediction (9 tests)<br>Compare within-domain feature-transfer against between-domain feature transfer (6 tests)
Figure 8	Compare average PNF value of each network block for each brain state against chance-level PNF values (171 unique blocks * 4 brain states * 3 behavioral domain = 2052 tests)
Supplementary Figure 14	Compare average PNF value of each network block for each brain state against chance-level PNF values (171 unique blocks * 4 brain states * 3 behavioral domain = 2052 tests)
**Third set of statistical corrections (total of 36 statistical tests)**	
Supplementary Table 4	Compare behavioral distributions of included and excluded participants (36 tests)
**Fourth set of statistical corrections (total of 180 statistical tests)**	
Supplementary Table 9	Compare distributions of age, sex, race, income, and behavior between included and excluded participants (5 variables matched * 36 behaviors = 180 tests)

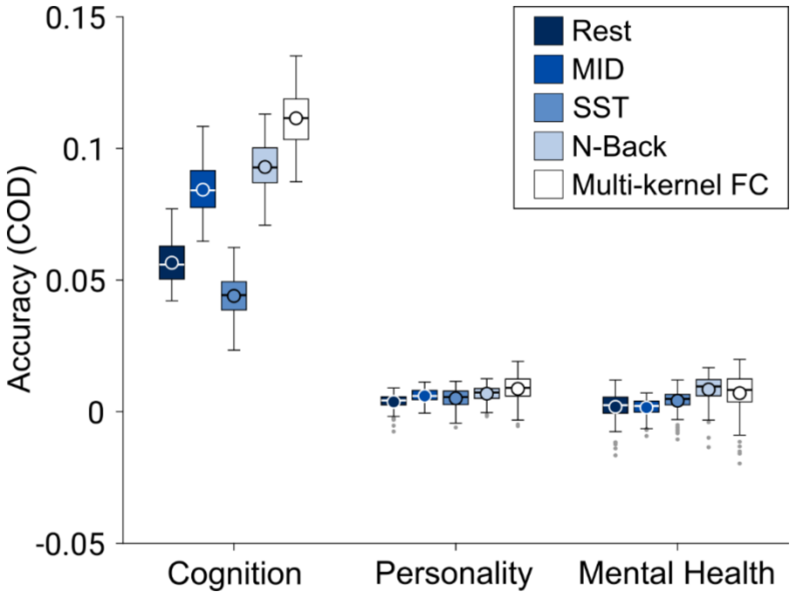

Supplementary Figure 1. Cross-validated prediction performance (coefficient of determination; COD) using kernel ridge regression for resting-state and task-states (MID, SST, N-Back). Multi-kernel FC utilized FC from all 4 brain states for prediction. Higher COD indicates greater variance predicted relative to the mean of the training data. No statistical test was performed here. Source data are provided as a Source Data file.

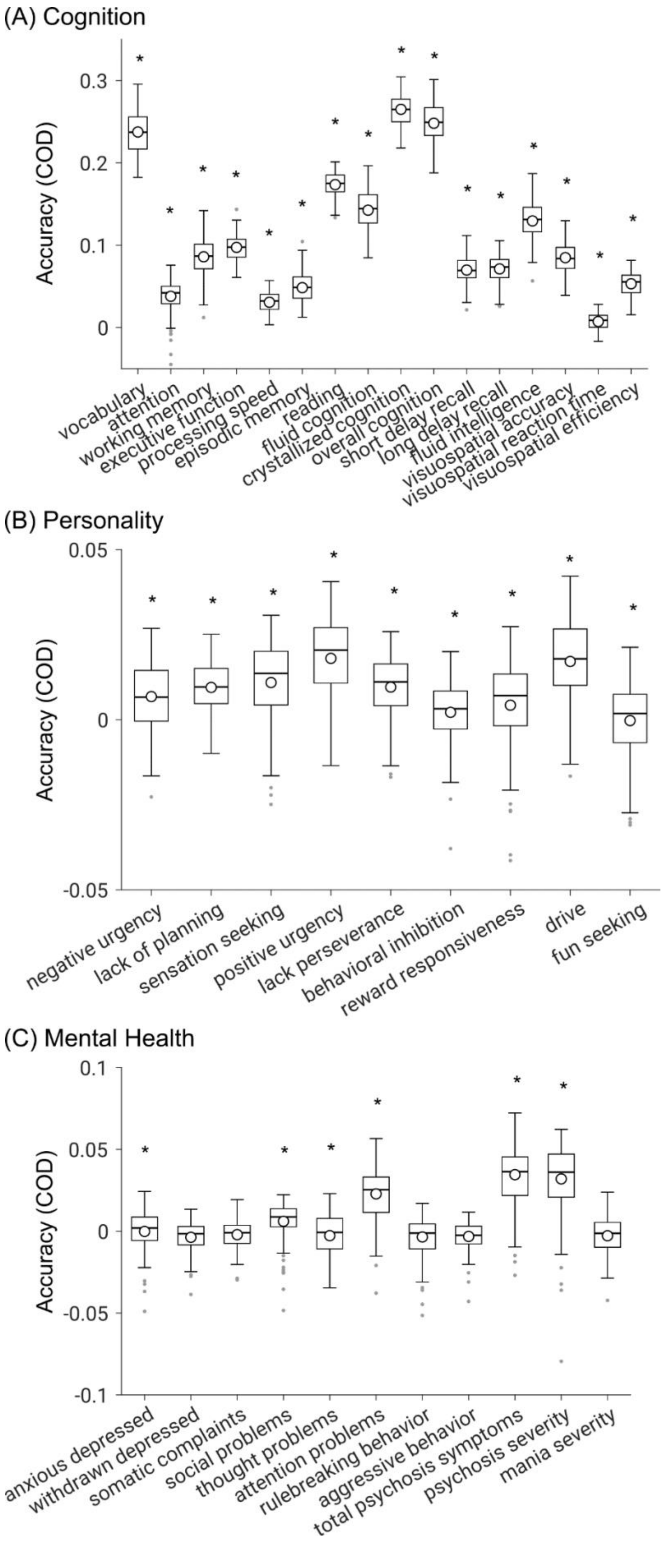
(A) Cognition
Accuracy (COD)
0.3
0.2
0.1
0
vocabulary
attention
working memory
executive function
processing speed
episodic memory
reading
fluid cognition
crystallized cognition
overall cognition
short delay recall
long delay recall
fluid intelligence
visuospatial accuracy
visuospatial reaction time
visuospatial efficiency
(B) Personality
Accuracy (COD)
0.05
0
-0.05
negative urgency
lack of planning
sensation seeking
positive urgency
lack perseverance
behavioral inhibition
reward responsiveness
drive
fun seeking
(C) Mental Health
Accuracy (COD)
0.1
0.05
0
-0.05
-0.1
anxious depressed
withdrawn depressed
somatic complaints
social problems
thought problems
attention problems
rulebreaking behavior
aggressive behavior
total psychosis symptoms
psychosis severity
mania severity

Supplementary Figure 2. Cross-validated prediction performance (coefficient of determination; COD) using multi-kernel ridge regression by exploiting resting-FC, MID-FC, SST-FC and N-back-FC jointly. (A) Cognitive measures. (B) Personality measures. (C) Mental health measures. * denotes above chance prediction after correcting for multiple comparisons (FDR $q < 0.05$). Source data are provided as a Source Data file.

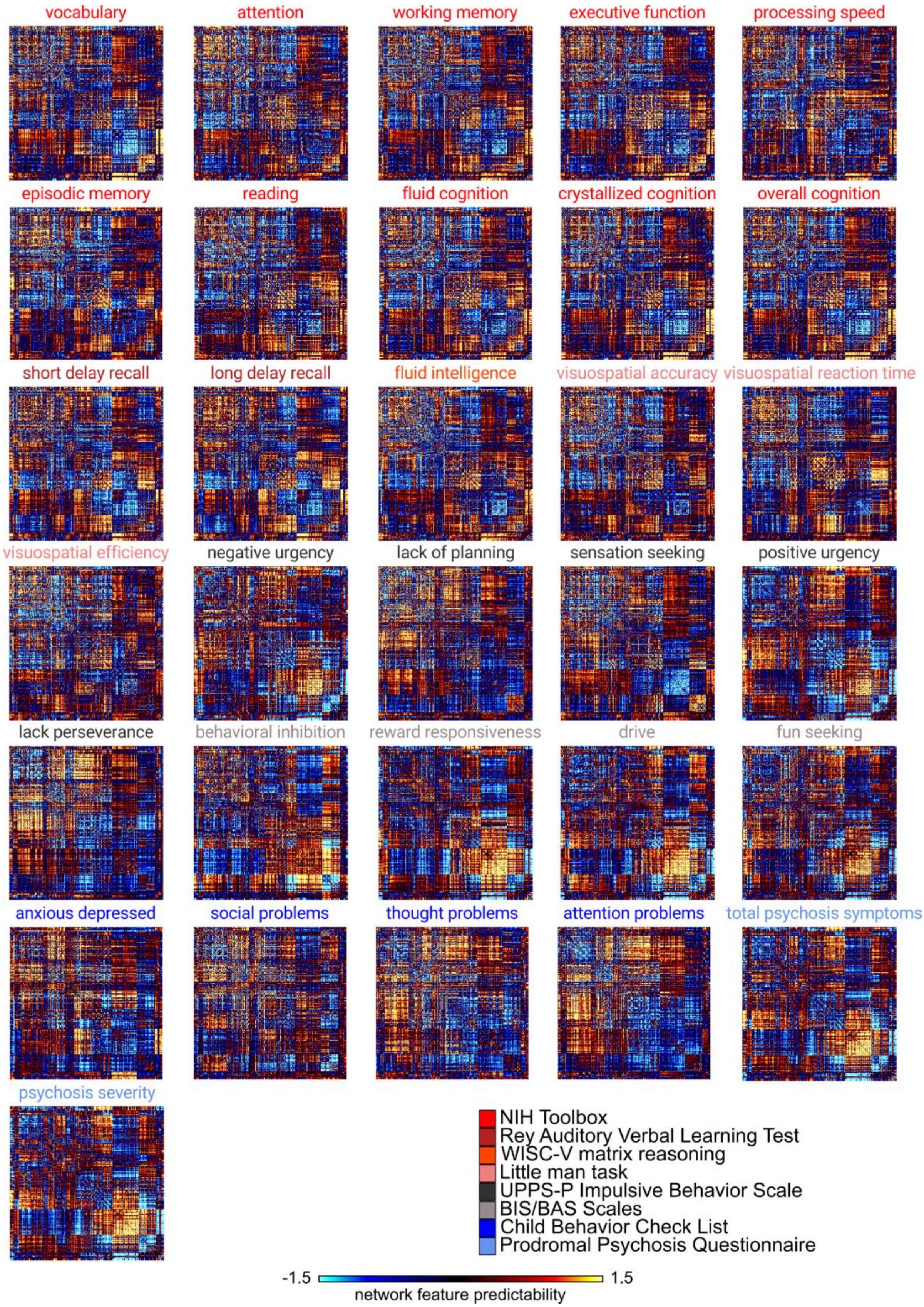

Supplementary Figure 3. Resting-FC predictive-feature matrices for each significantly predicted behavior. For visualization, the values within each matrix were divided by their standard deviations.

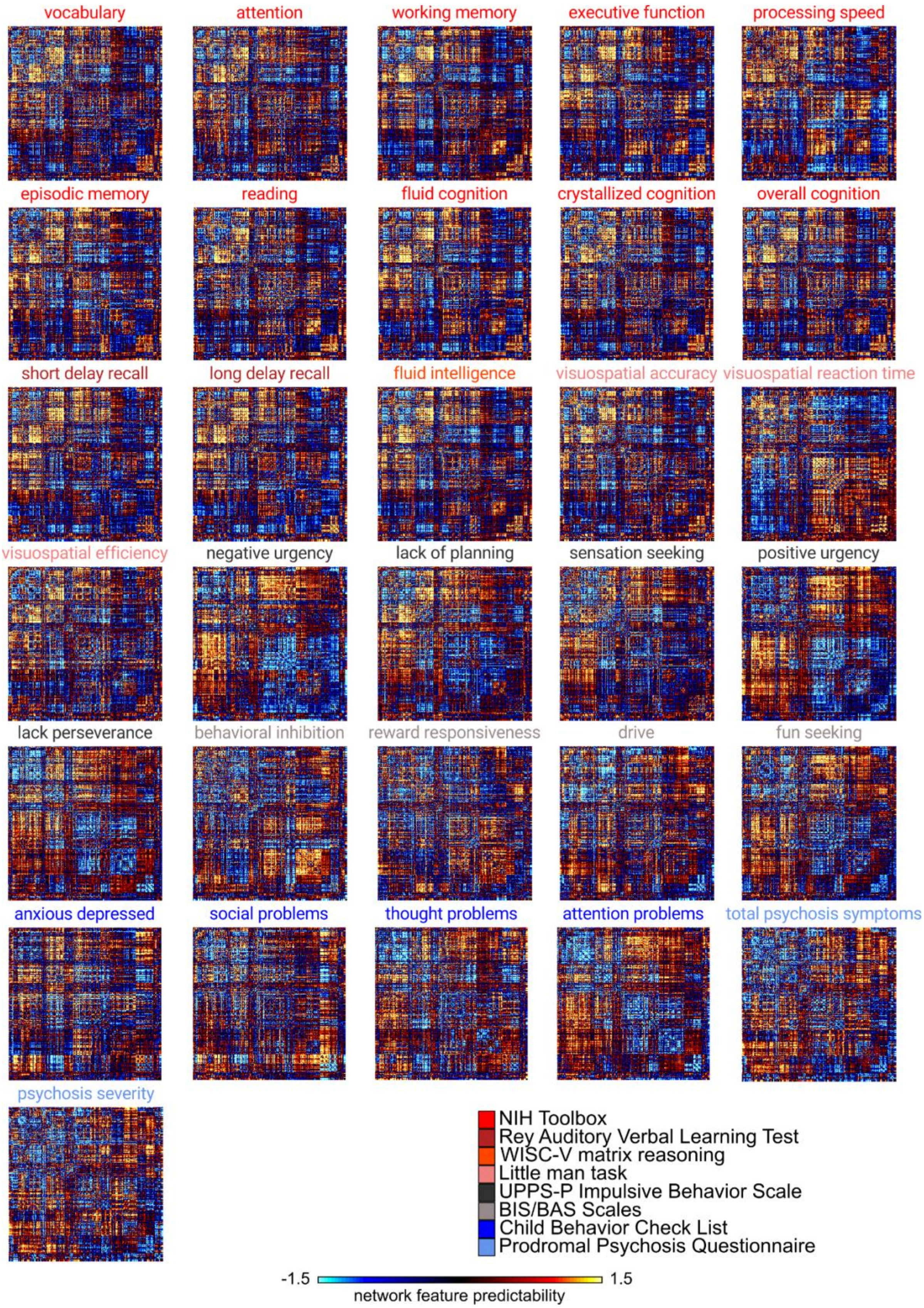

Supplementary Figure 4. MID-FC predictive-feature matrices for each significantly predicted behavior. For visualization, the values within each matrix were divided by their standard deviations.

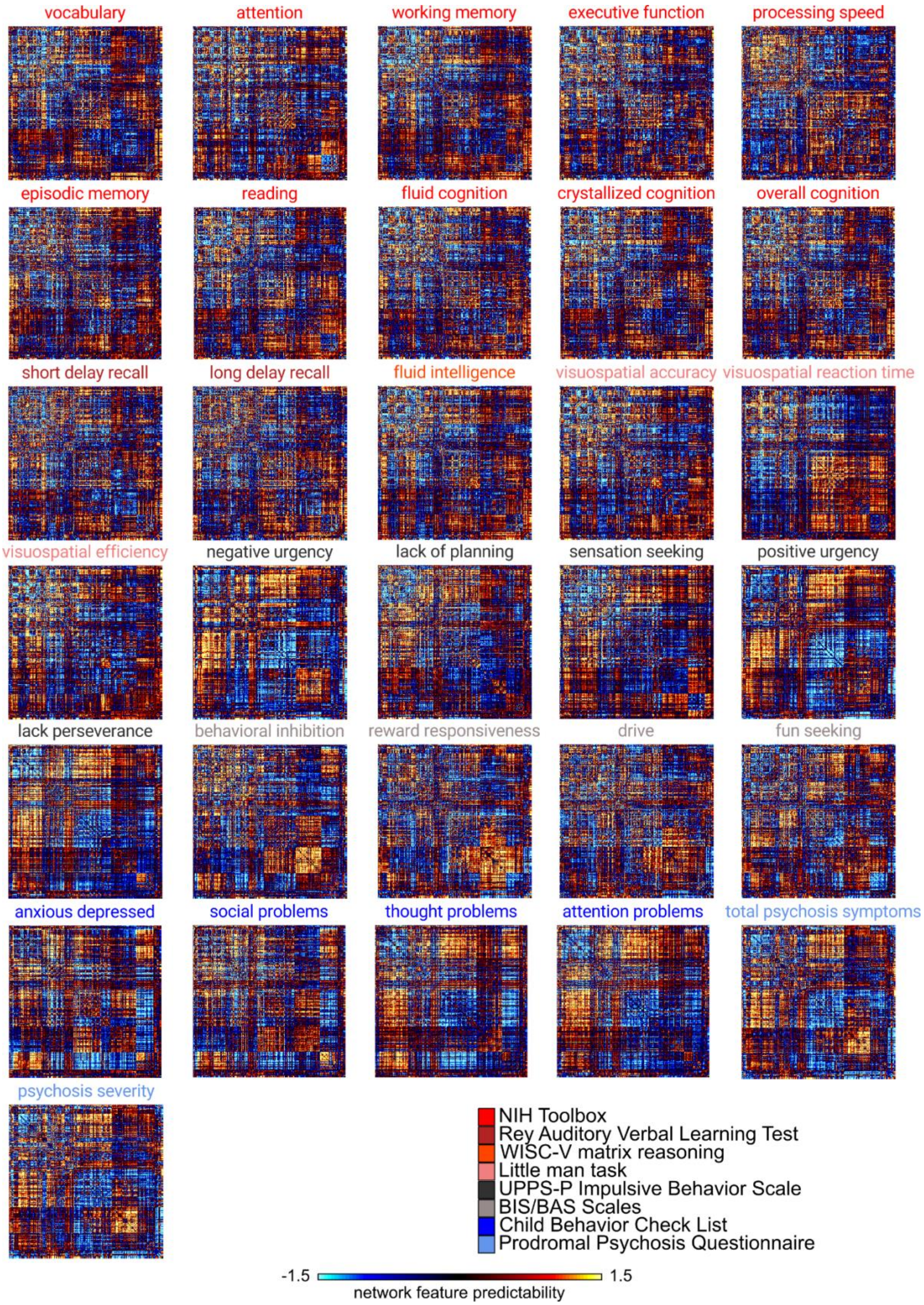

Supplementary Figure 5. SST-FC predictive-feature matrices for each significantly predicted behavior. For visualization, the values within each matrix were divided by their standard deviations.

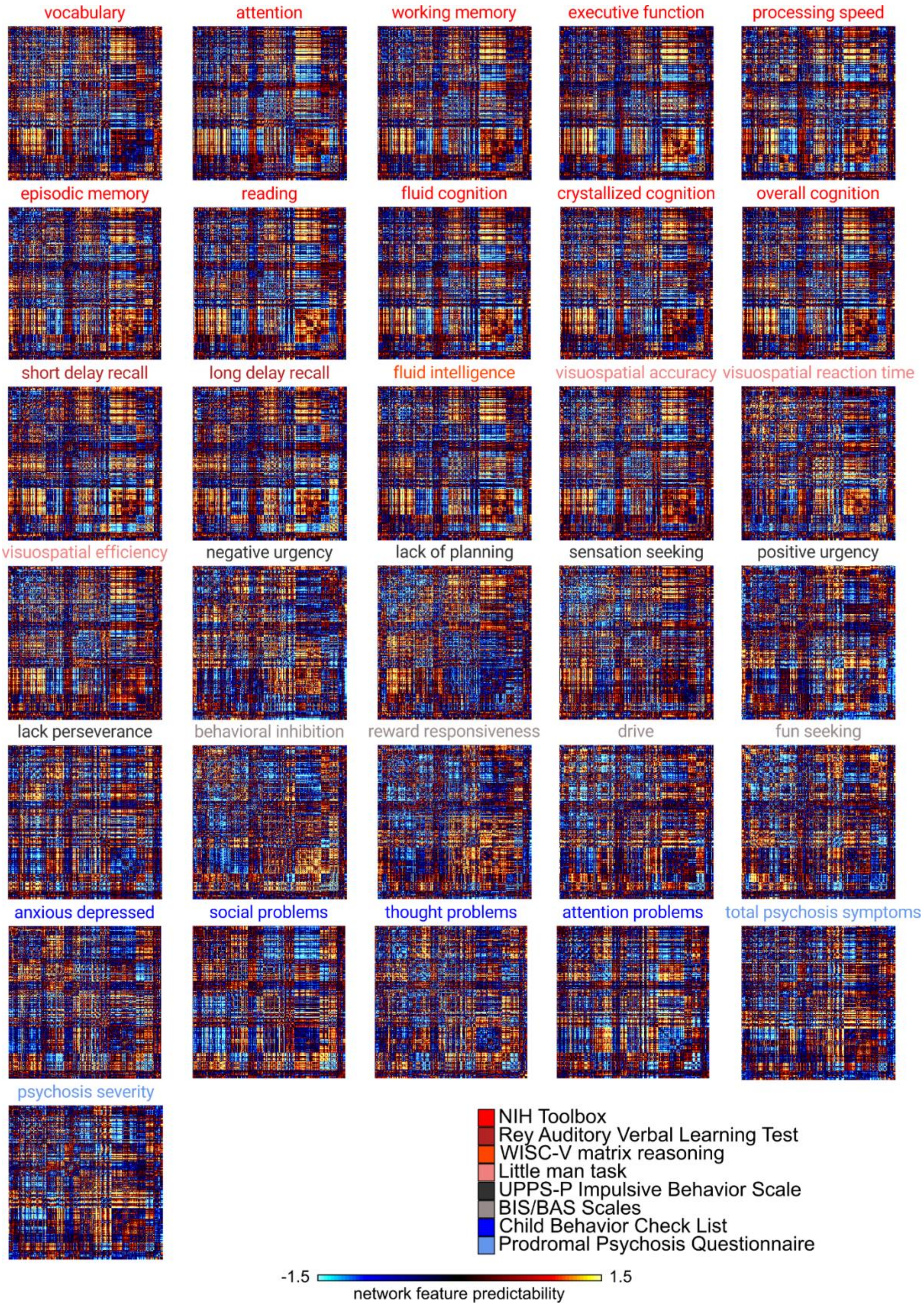

Supplementary Figure 6. N-Back-FC predictive-feature matrices for each significantly predicted behavior. For visualization, the values within each matrix were divided by their standard deviations.

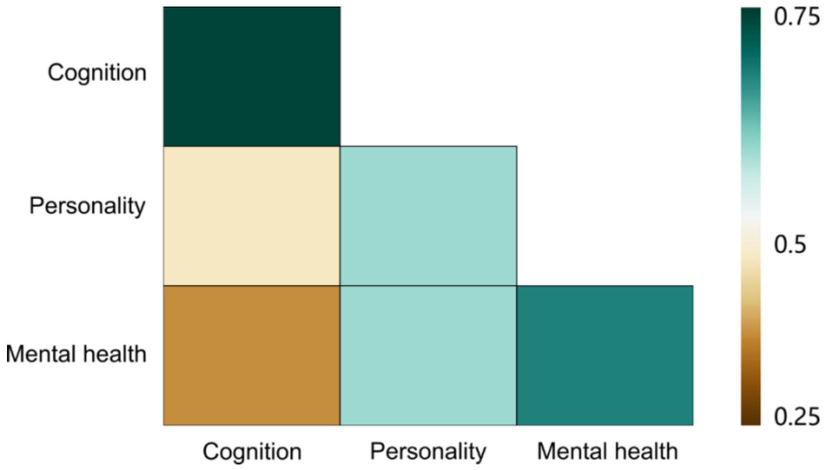

Supplementary Figure 7. Proportion of network blocks with consistent predictive network features. For each pair of behavioral measures, we computed the proportion of network blocks for which the predictive network features exhibited consistent directionality (positive or negative) across the two behavioral measures. Green indicates consistency greater than 50%, while brown indicates consistency less than 50%. Within each behavioral domain, the proportion of consistent predictive network features was significantly better than chance: 74% for cognition (p=6e-45), 58% for personality (p=1e-10) and 67% for mental health (p=4e-14). Each within-domain proportion was also significantly greater than the corresponding between-domain proportions ($p < 0.015$). The sole exception was the relatively high between-domain proportion for mental health and personality, consistent with Figure 4A. Source data are provided as a Source Data file.

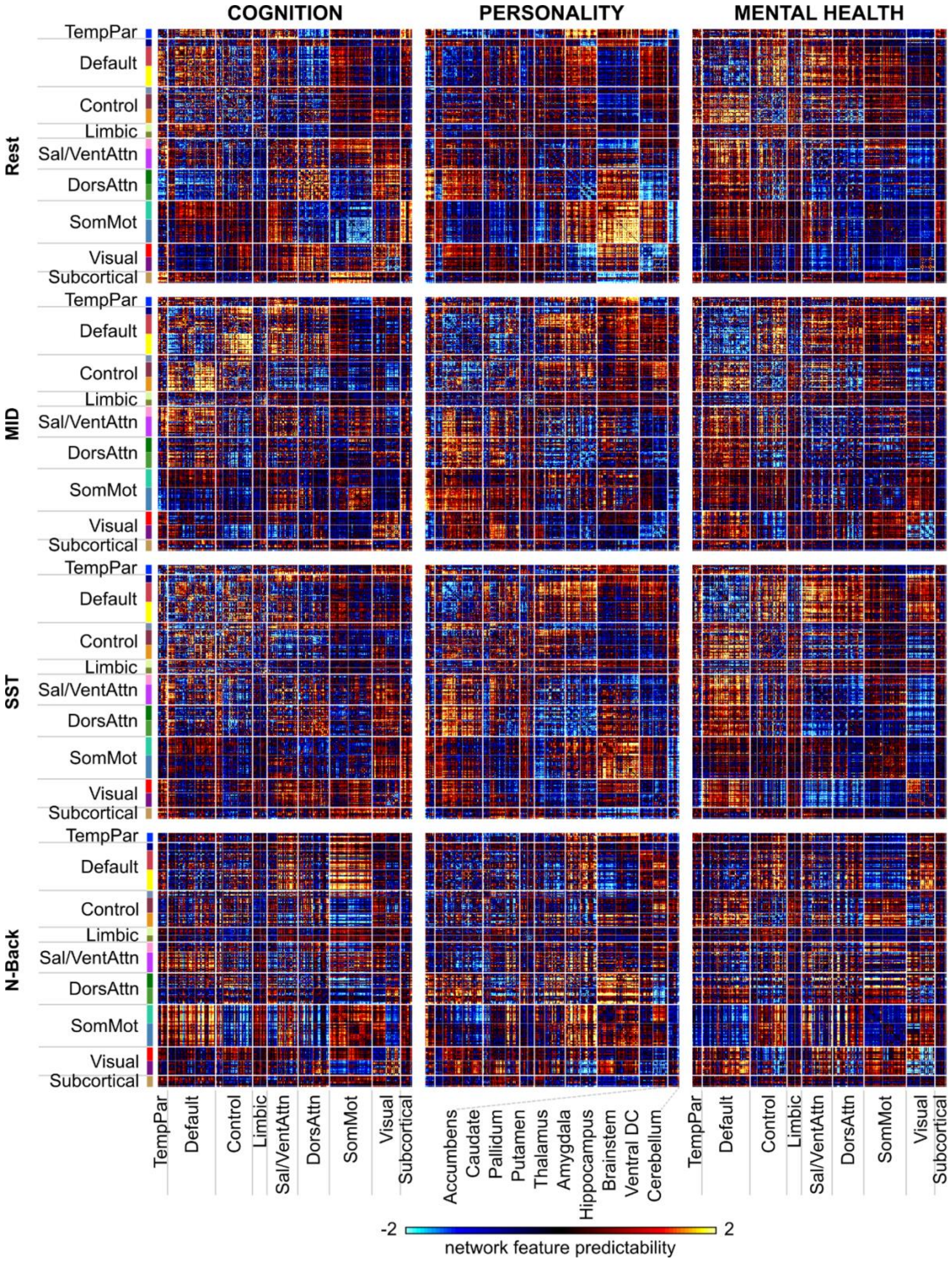

Supplementary Figure 8. Predictive-feature matrices for each brain state (Rest, MID, SST, N-Back) averaged across all behavioral measures within each data-driven behavioral cluster (cognition, personality, mental health). For visualization, the values within each matrix were divided by their standard deviations.

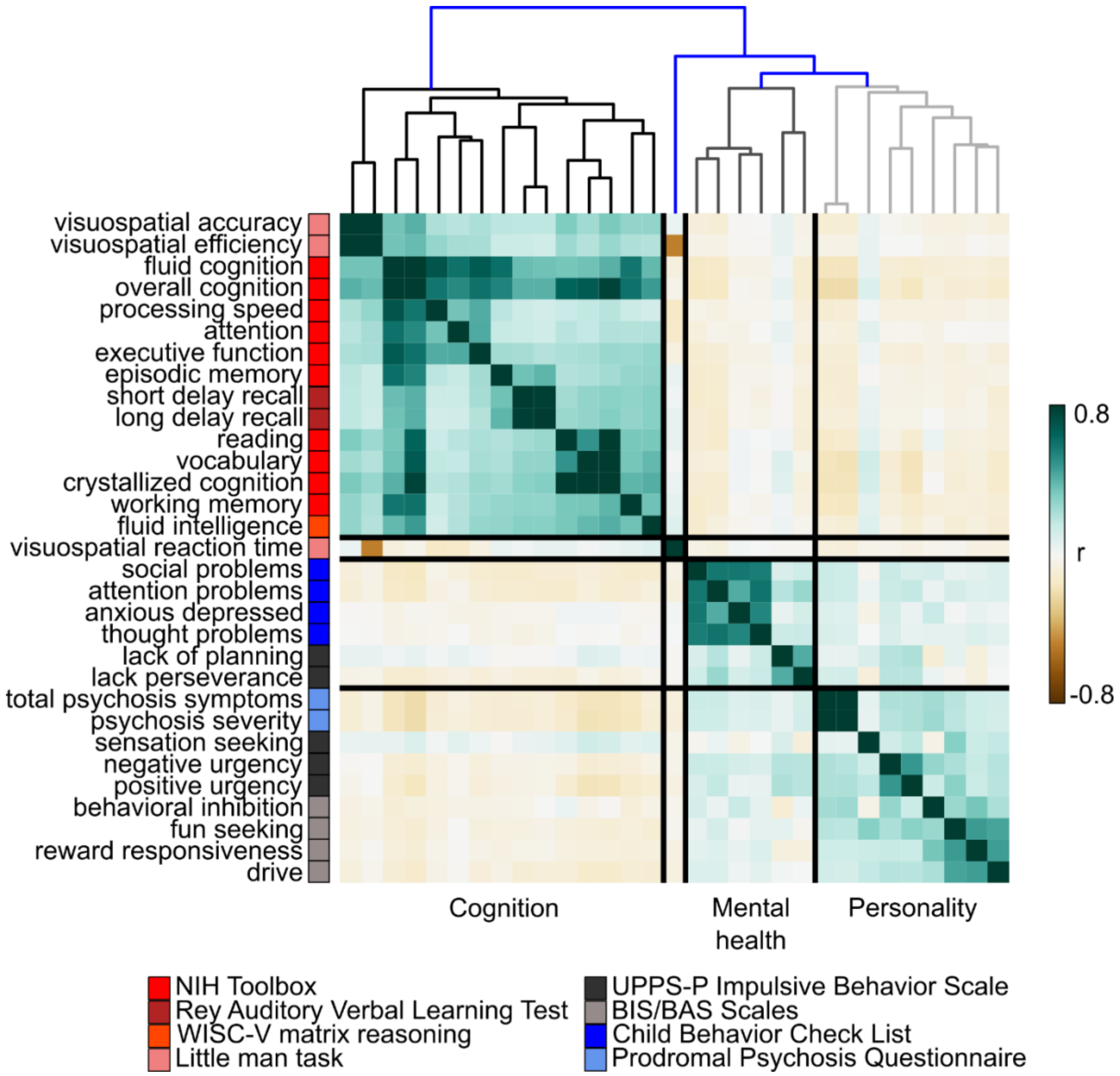

Supplementary Figure 9. Hierarchical clustering of actual behavioral scores. Clustering was performed using hierarchical agglomerative average linkage (UPGMA) clustering as implemented in scipy 1.2.1 (Virtanen *et al.* 2020).

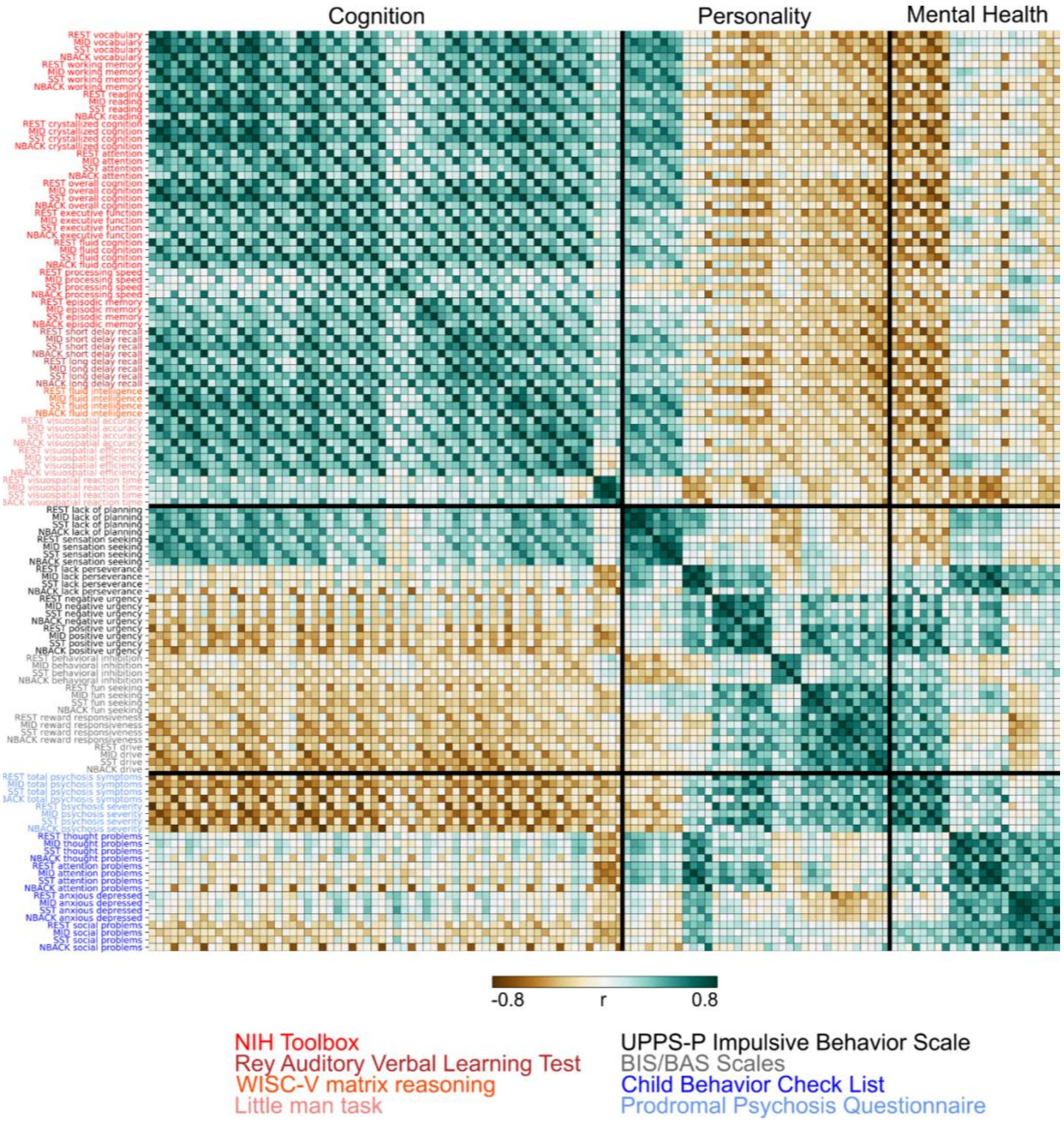

Supplementary Figure 10. Similarity of predictive-network features for each significantly predicted behavior and brain state. The behavioral measures were ordered based on hypothesis-driven behavioral domains (cognition, personality and mental health). For each behavior, the brain states were ordered by Rest, MID, SST and finally N-Back. Red font indicates cognitive measures. Black/grey font indicates personality measures. Blue font indicates mental health measures. Predictive-network features were highly correlated within each hypothesis-driven behavioral domain and across brain states.

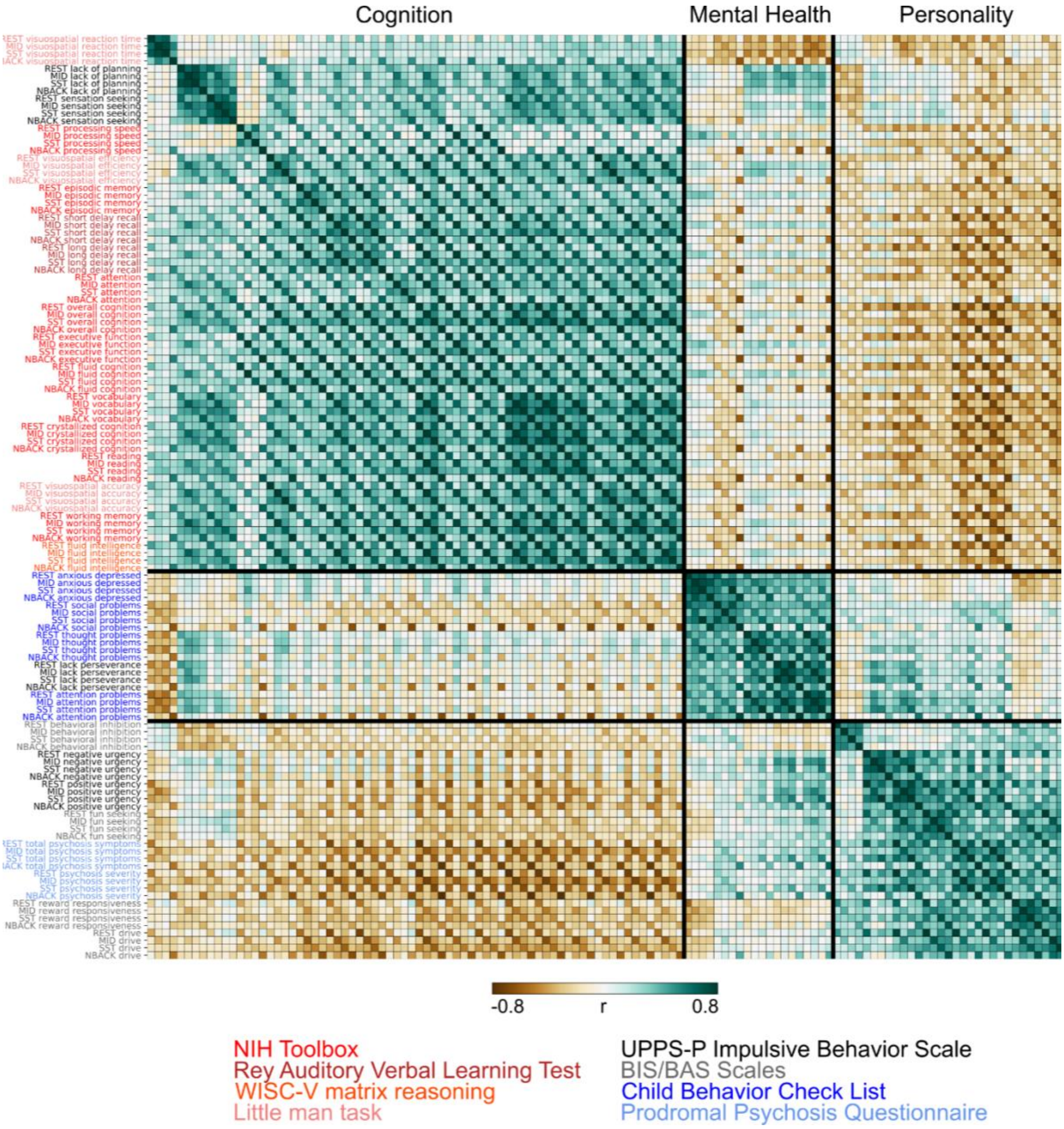

Supplementary Figure 11. Similarity of predictive-network features for each significantly predicted behavior and brain state. The behavioral measures were ordered based on data-driven behavioral clusters (cognition, personality and mental health). For each behavior, the brain states were ordered by Rest, MID, SST and finally N-Back. Red font indicates cognitive measures. Black/grey font indicates personality measures. Blue font indicates mental health measures. Predictive-network features were highly correlated within each hypothesis-driven behavioral domain and across brain states.

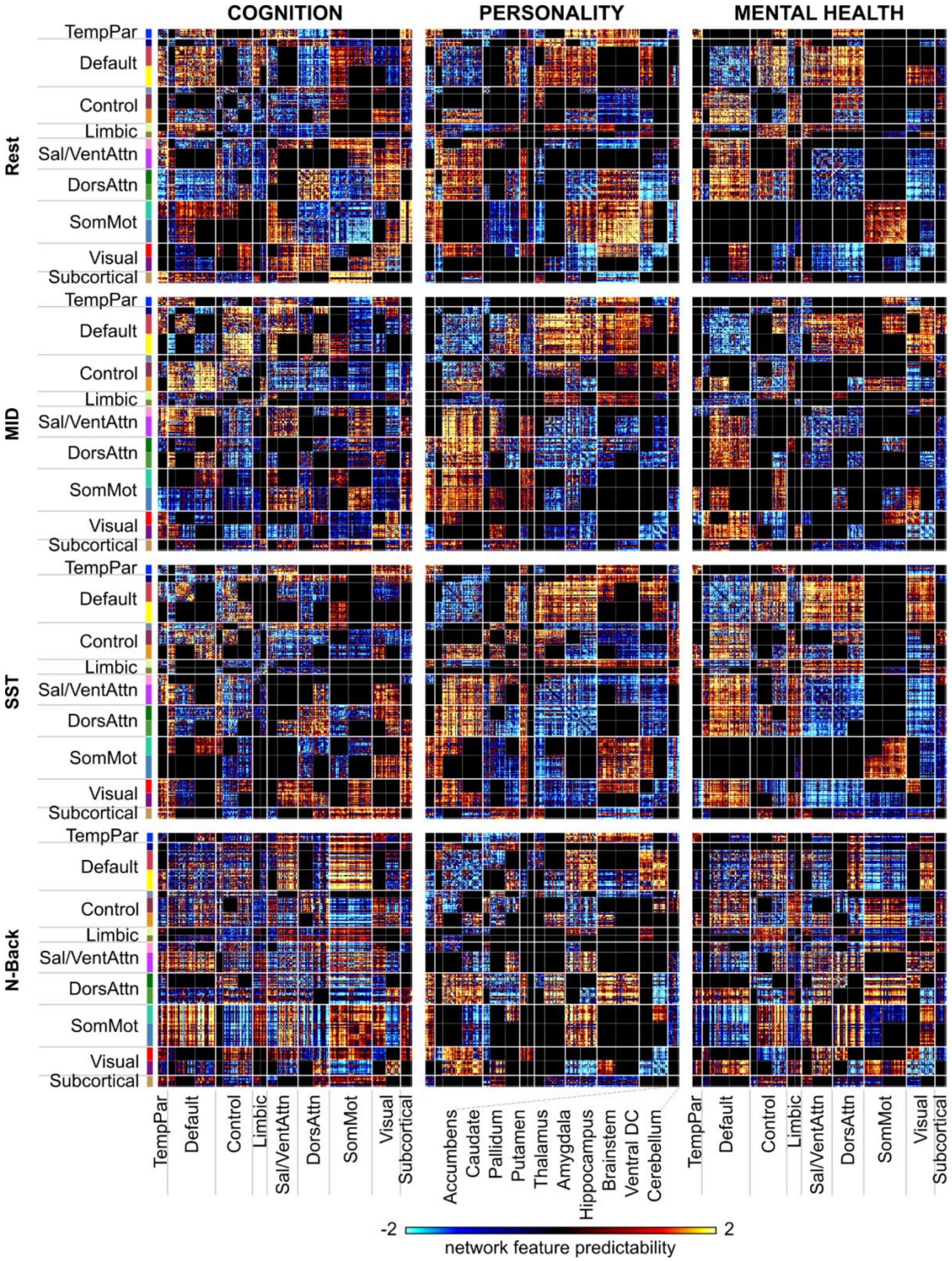

Supplementary Figure 12. Predictive-feature matrices showing significant network blocks for each hypothesis-driven behavioral domain (cognitive, personality, mental health) for each brain state (Rest, MID, SST, N-Back) after permutation testing. For visualization, the values within each matrix were divided by their standard deviations.

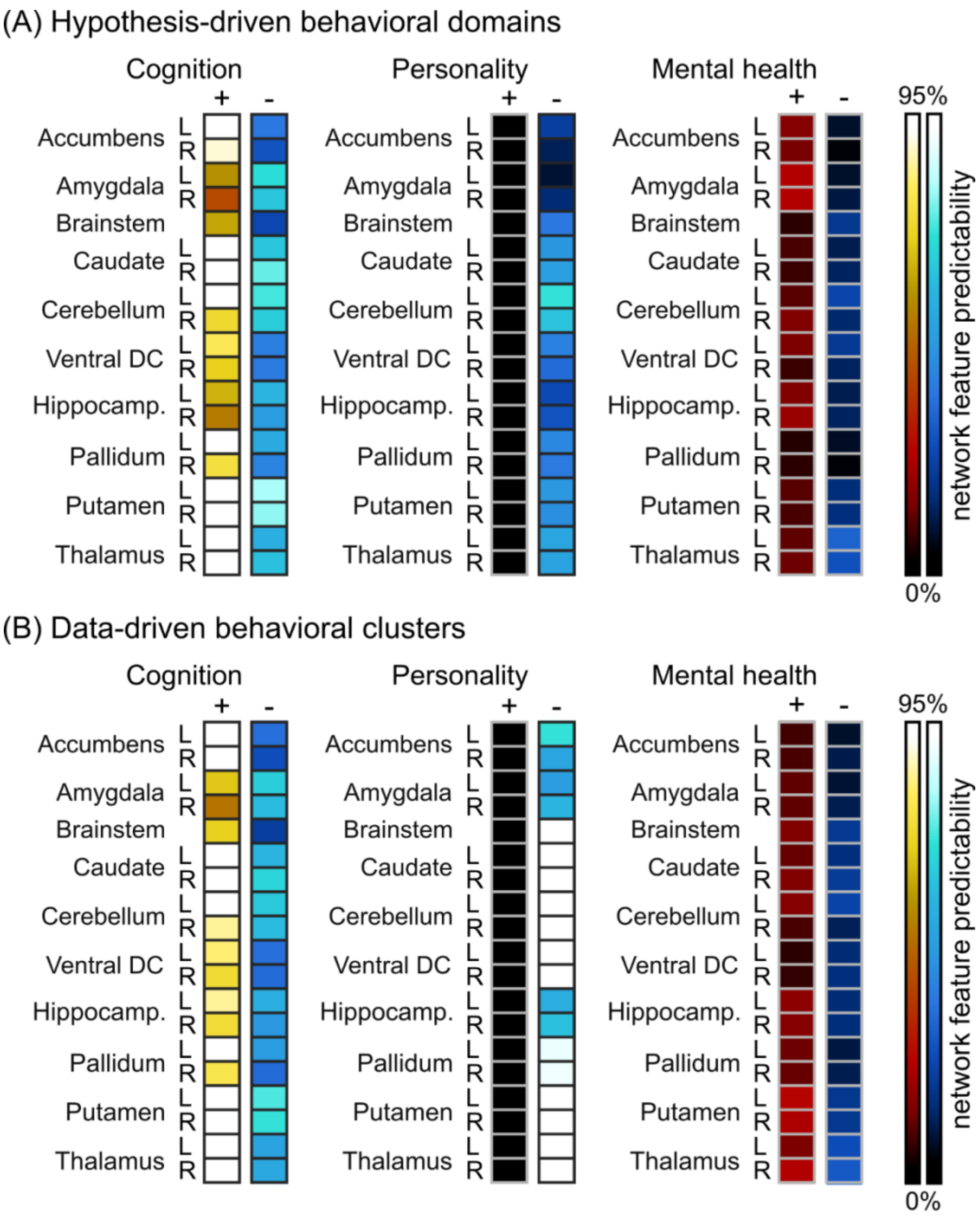

Supplementary Figure 13. Heatmaps showing network feature predictability of each subcortical region for (A) each hypothesis-driven behavioral domain and (B) each data-driven behavioral cluster. See Figures 8C and 8D for the cortical maps of the hypothesis-driven behavioral domains and Supplementary Figures 15C and 15D for the cortical maps of the data-driven behavioral clusters.

(A) Cognition

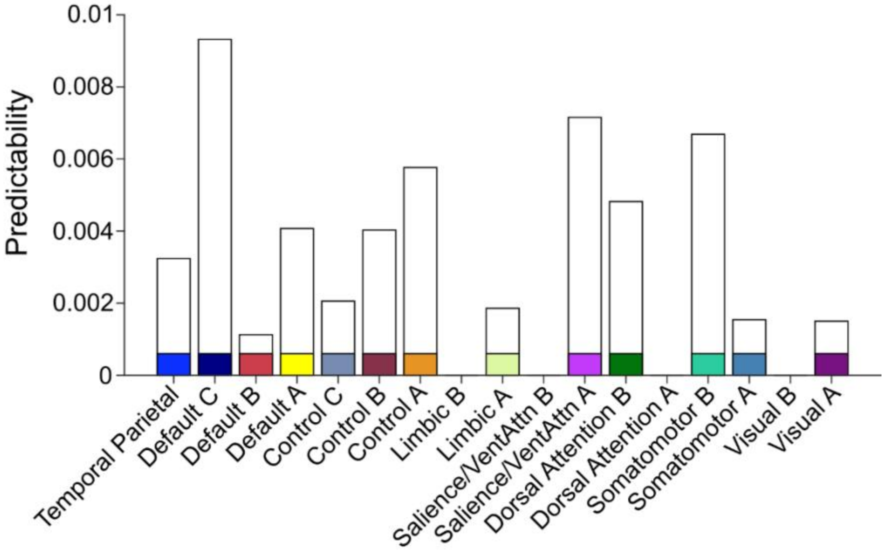

(B) Personality

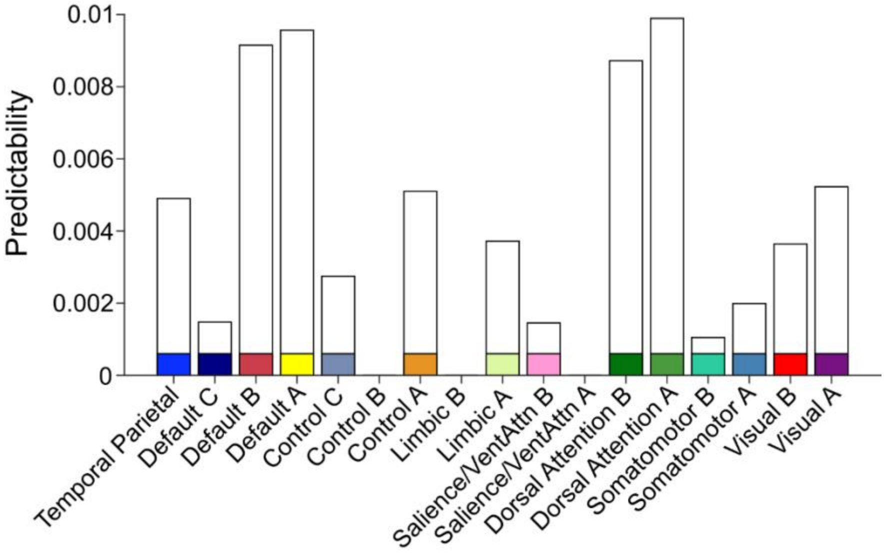

(C) Mental Health

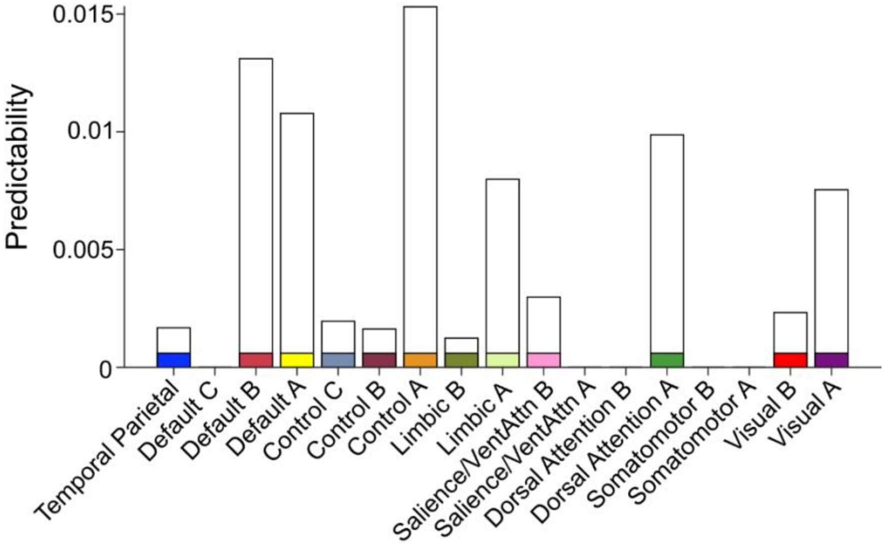

Supplementary Figure 14. Bar plots showing average predictive-network feature values for the hypothesis-driven domains of (A) cognition, (B) personality and (C) personality behaviors. For each network, average predictive-network feature values were obtained by averaging the absolute predictive-network feature values of all brain regions within the network in Figures 8C and 8D. Color in the bar plots corresponds to network color (Figure 1B).

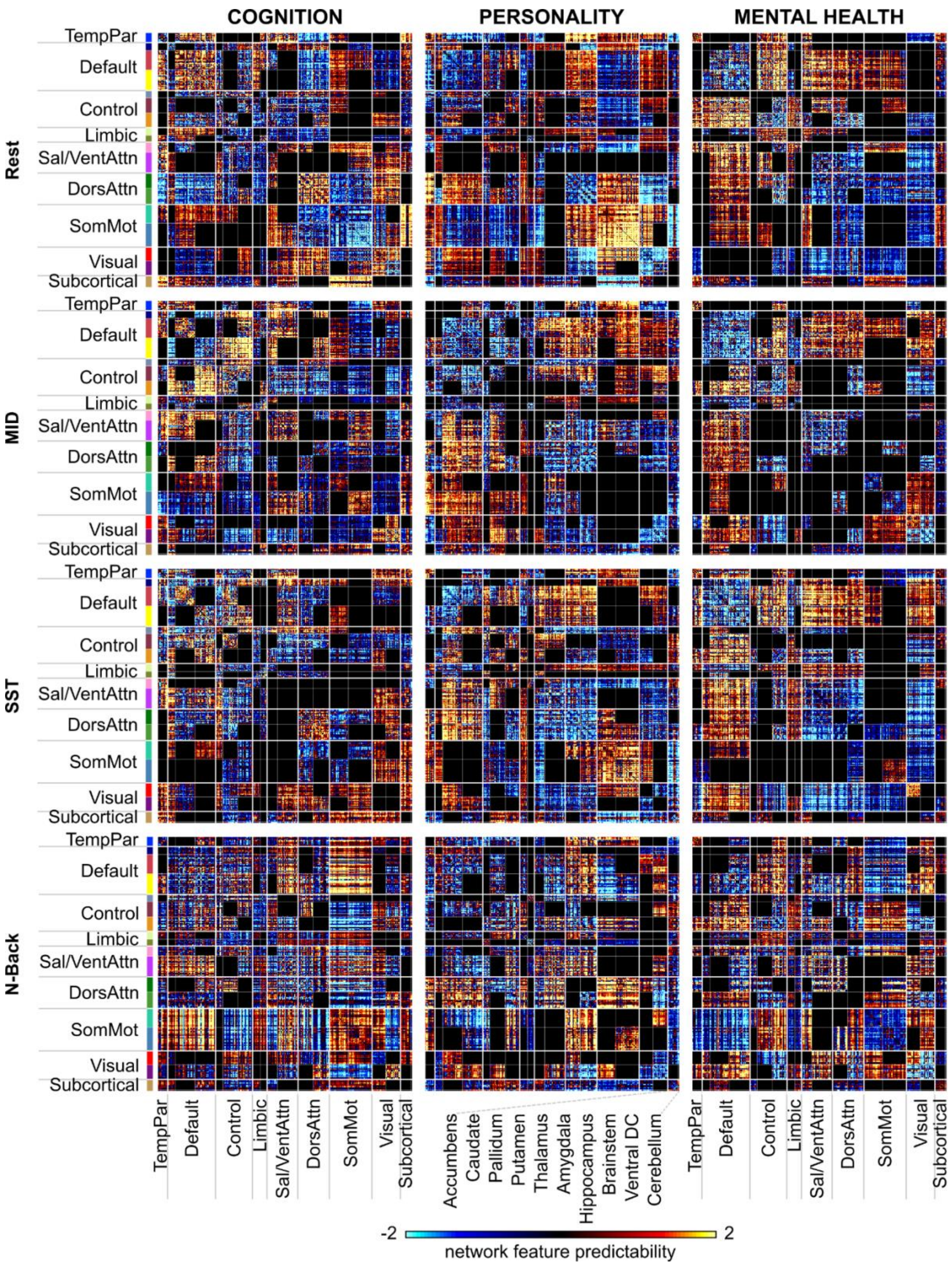

Supplementary Figure 15. Predictive-feature matrices showing significant network blocks for each data-driven behavioral cluster (cognitive, personality, mental health) and for each brain state (Rest, MID, SST, N-Back) after permutation testing. For visualization, the values within each matrix were divided by their standard deviations.

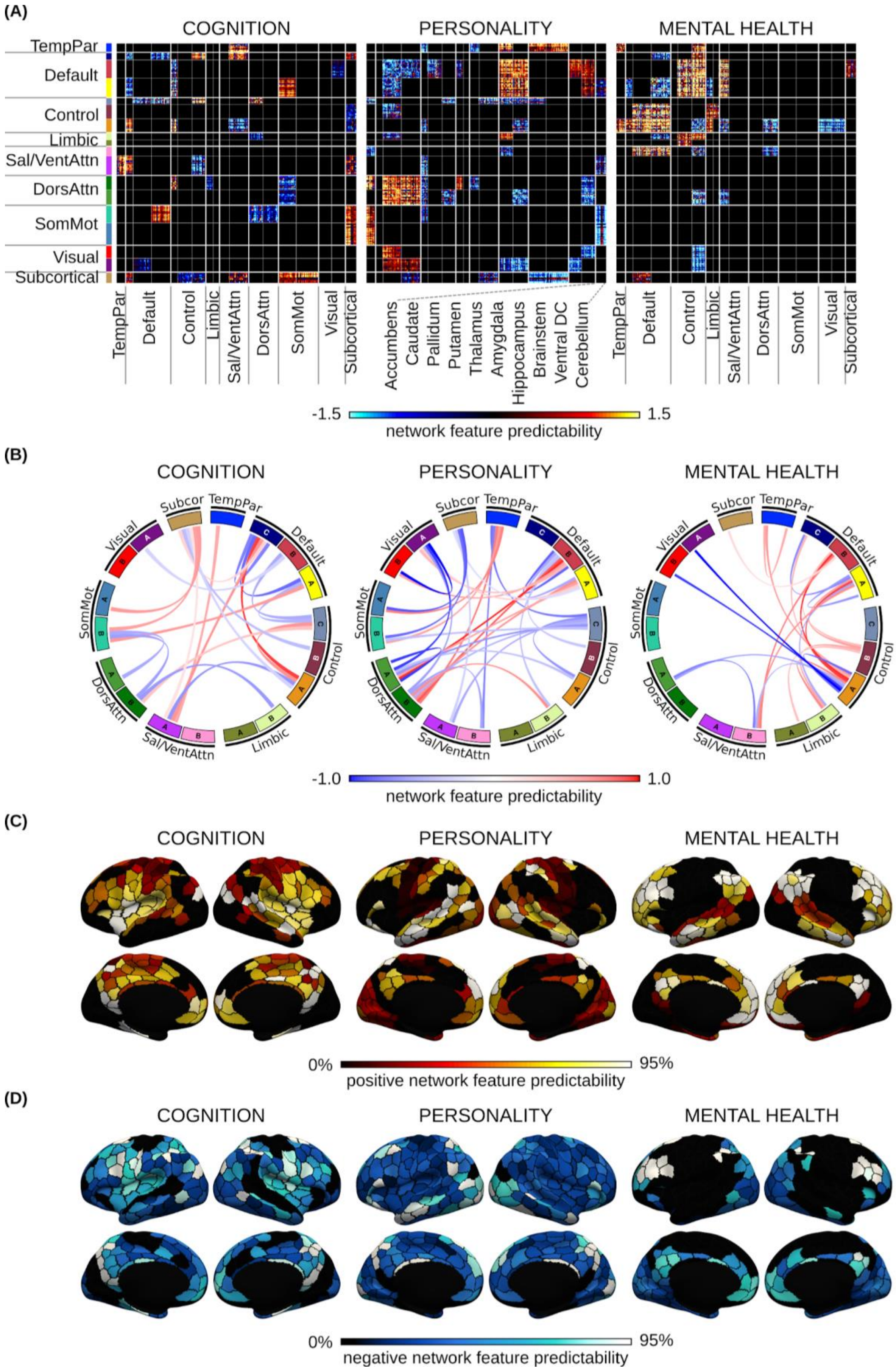
(A)
COGNITION
PERSONALITY
MENTAL HEALTH
TempPar
Default
Control
Limbic
Sal/VentAttn
DorsAttn
SomMot
Visual
Subcortical
Accumbens
Caudate
Pallidum
Putamen
Thalamus
Amygdala
Hippocampus
Brainstem
Ventral DC
Cerebellum
-1.5
1.5
network feature predictability
(B)
COGNITION
PERSONALITY
MENTAL HEALTH
Subcor
TempPar
Default
Control
Limbic
Sal/VentAttn
DorsAttn
SomMot
Visual
-1.0
1.0
network feature predictability
(C)
COGNITION
PERSONALITY
MENTAL HEALTH
0%
95%
positive network feature predictability
(D)
COGNITION
PERSONALITY
MENTAL HEALTH
0%
95%
negative network feature predictability

Supplementary Figure 16. Predictive brain network features for predicting cognition, personality and mental health. This figure is the same as Figure 8 but using data-driven behavioral clusters, instead of hypothesis-driven behavioral domains. (A) Predictive-feature matrices averaged across brain states, considering only within-network and between-network blocks that were significant across all four brain states (Rest, MID, SST, N-Back). (B) Predictive network connections obtained by averaging the matrices in panel (A) within each between-network and within-network block. (C) Positive predictive features obtained by summing positive predictive-feature values across the rows of panel (A). A higher value for a brain region indicates that stronger connectivity yielded a higher prediction for the behavioral measure. (D) Negative predictive features obtained by summing negative predictive-feature values across the rows of panel (A). A higher value for a brain region indicates that weaker connectivity yielded a greater prediction for the behavioral measure. Conclusions were highly similar using hypothesis-driven behavioral domains (Figure 8).

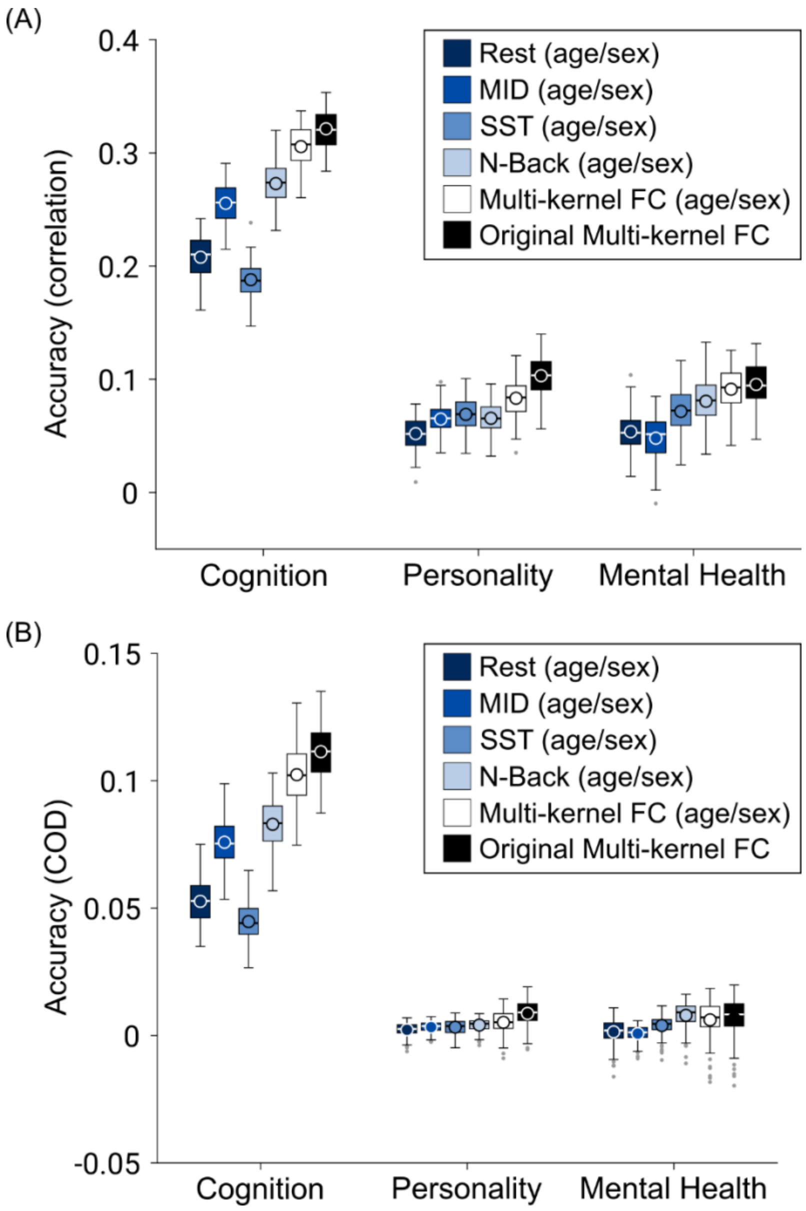

Supplementary Figure 17. Mean cross-validated prediction performance after regressing out age and sex from the behaviors, compared to the prediction performance of the original multi-kernel FC regression model (as shown in main text) without the regression of age and sex. (A) Accuracy as measured by Pearson's correlation between observed and predicted values. (B) Accuracy as measured by the coefficient of determination (COD). Source data are provided as a Source Data file.

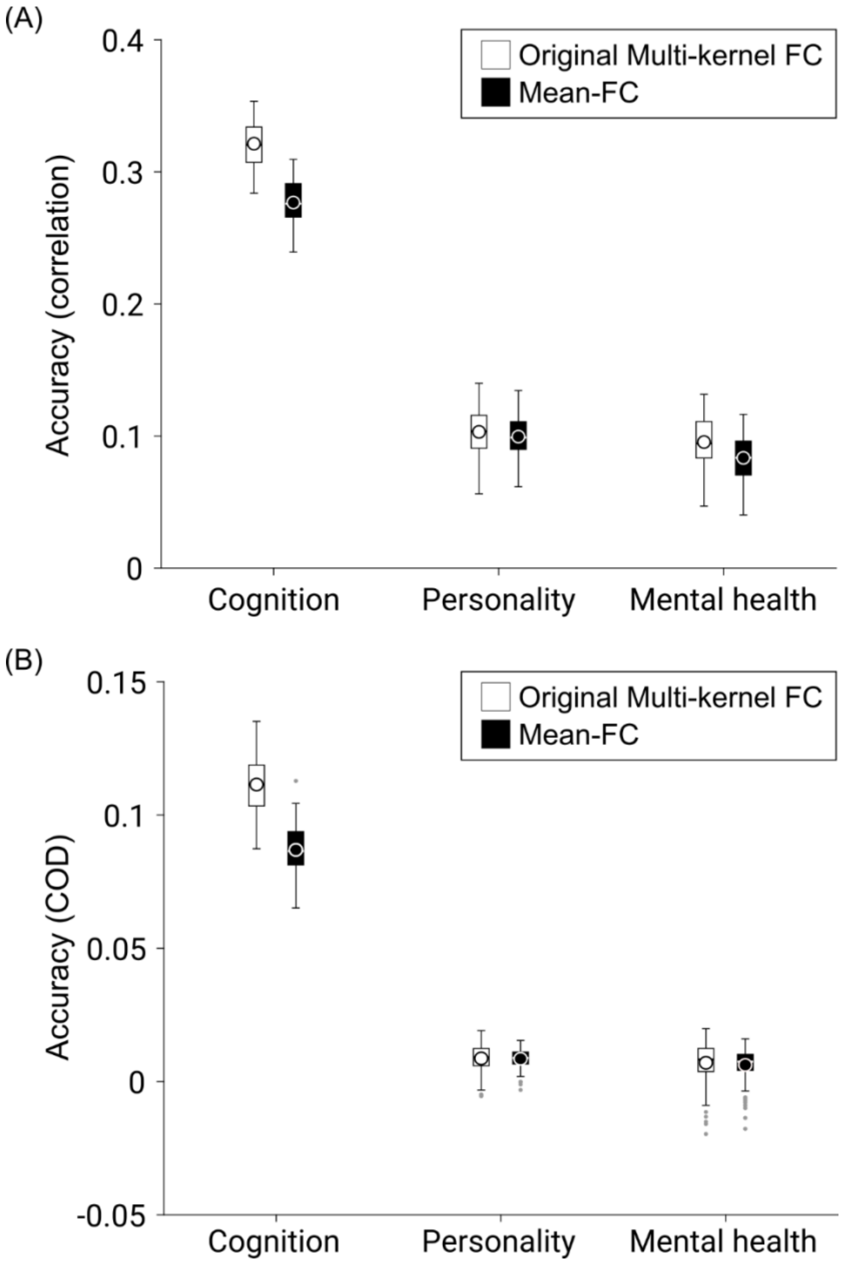

Supplementary Figure 18. Mean cross-validated prediction performance obtained by the original multi-kernel FC regression model (as shown in main text) and kernel ridge regression using FC averaged across all four brain states (mean-FC). (A) Accuracy as measured by Pearson's correlations between observed and predicted values. (B) Accuracy as measured by the coefficient of determination (COD). Source data are provided as a Source Data file.

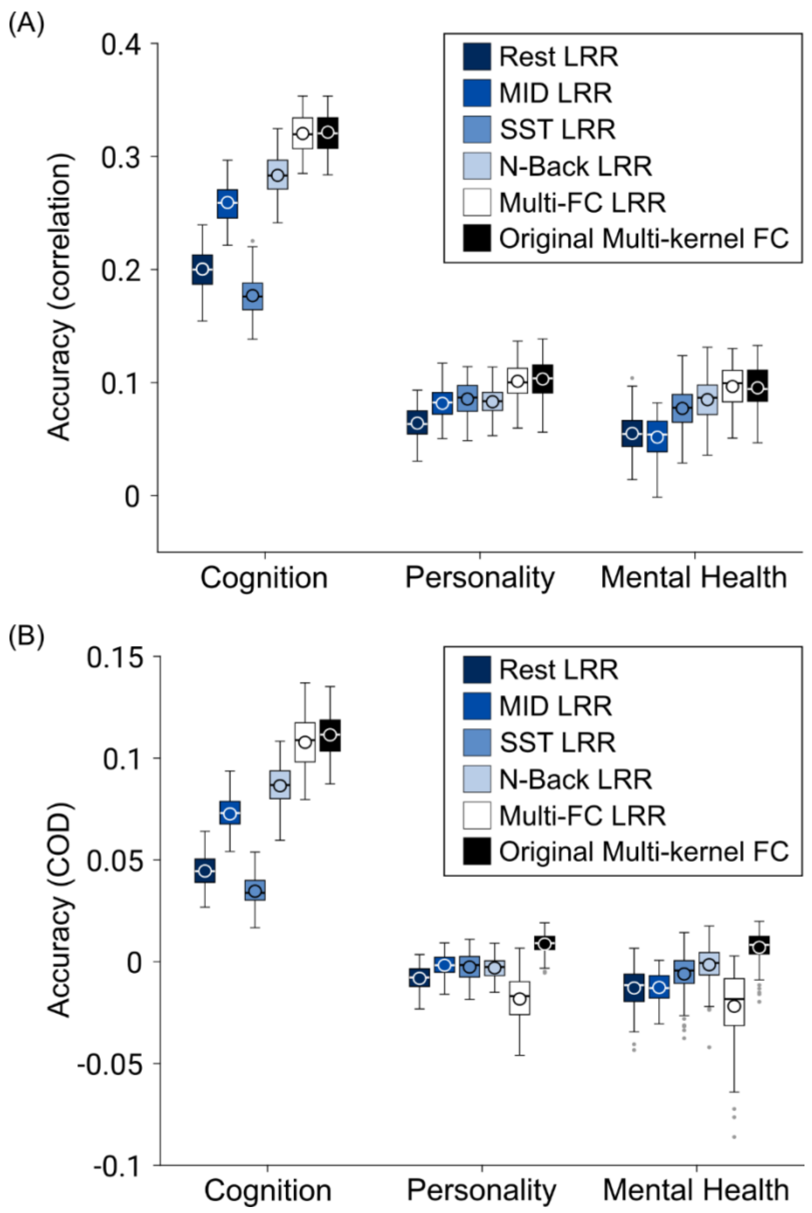

Supplementary Figure 19. Mean cross-validated prediction performance using linear ridge regression (LRR) and the original multi-kernel FC regression model (as shown in main text). (A) Accuracy as measured by Pearson's correlations between observed and predicted values. (B) Accuracy as measured by the coefficient of determination (COD). Source data are provided as a Source Data file.

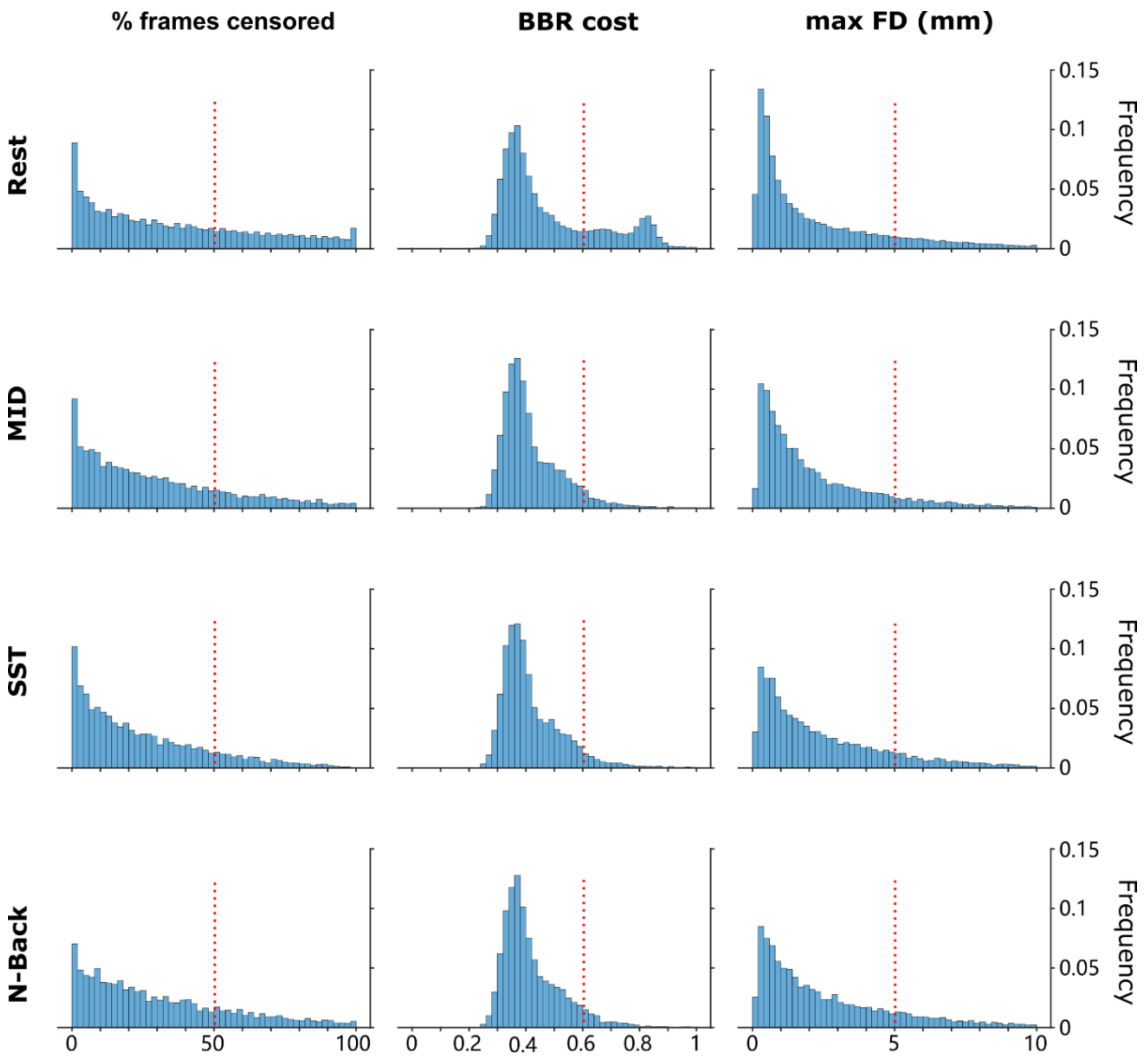

Supplementary Figure 20. Distribution of QC metrics. BBR refers to boundary-based registration. A larger BBR cost indicates worse alignment between anatomical and functional images. Dotted red lines show the QC cut-off. Runs with more than 50% frames censored, BBR cost > 0.6 or max FD > 5mm were removed. Note that the rest runs were from 10,277 participants, while the task runs were from 4,506 participants (Figure 1A).

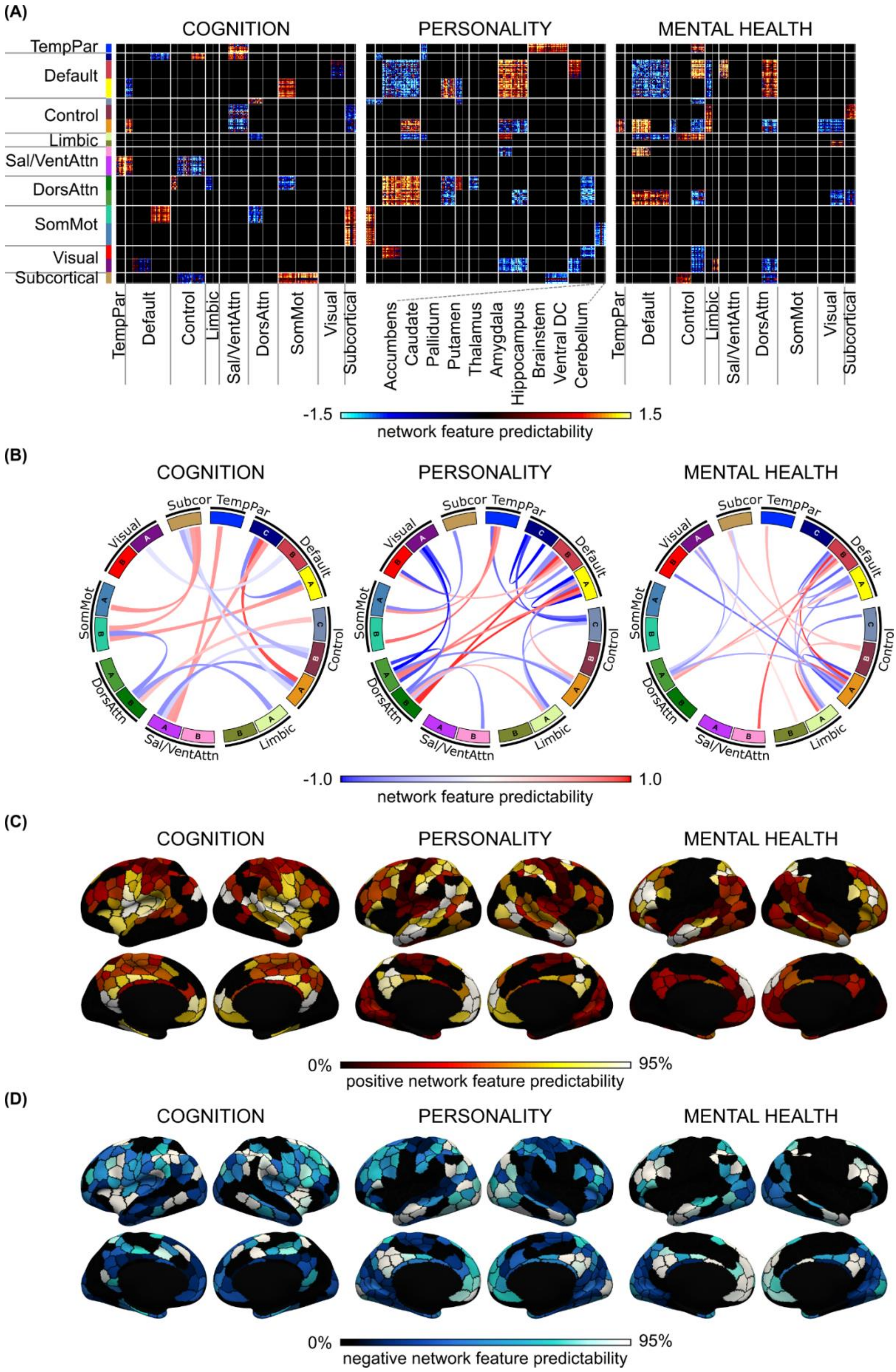

(A)
COGNITION
PERSONALITY
MENTAL HEALTH
TempPar
Default
Control
Limbic
Sal/VentAttn
DorsAttn
SomMot
Visual
Subcortical
Accumbens
Caudate
Pallidum
Putamen
Thalamus
Amygdala
Hippocampus
Brainstem
Ventral DC
Cerebellum
-1.5
1.5
network feature predictability
(B)
COGNITION
PERSONALITY
MENTAL HEALTH
Subcor
TempPar
Default
Control
Limbic
Sal/VentAttn
DorsAttn
SomMot
Visual
-1.0
1.0
network feature predictability
(C)
COGNITION
PERSONALITY
MENTAL HEALTH
0%
95%
positive network feature predictability
(D)
COGNITION
PERSONALITY
MENTAL HEALTH
0%
95%
negative network feature predictability

Supplementary Figure 21. Brain network features that support individual-level prediction of cognition, personality and mental health. This figure is the same as Figure 8 but using data processed with respiratory pseudo-motion filtering (N = 2262). (A) Predictive-feature matrices averaged across brain states, considering only within-network and between-network blocks that were significant across all four brain states (Rest, MID, SST, N-Back). (B) Predictive network connections obtained by averaging the matrices in panel (A) within each between-network and within-network block. (C) Positive predictive features obtained by summing positive predictive-feature values across the rows of panel (A). A higher value for a brain region indicates that stronger connectivity yielded a higher prediction for the behavioral measure. (D) Negative predictive features obtained by summing negative predictive-feature values across the rows of panel (A). A higher value for a brain region indicates that weaker connectivity yielded a greater prediction for the behavioral measure. In both panels C and D, the color of each parcel corresponds to the percentile of predictive-feature values among 400 parcels. For visualization, the values within each predictive-feature matrix in panel A were divided by their standard deviations across all entries in the predictive-feature matrix. Conclusions were highly similar using data from original processing pipeline (Figure 8).

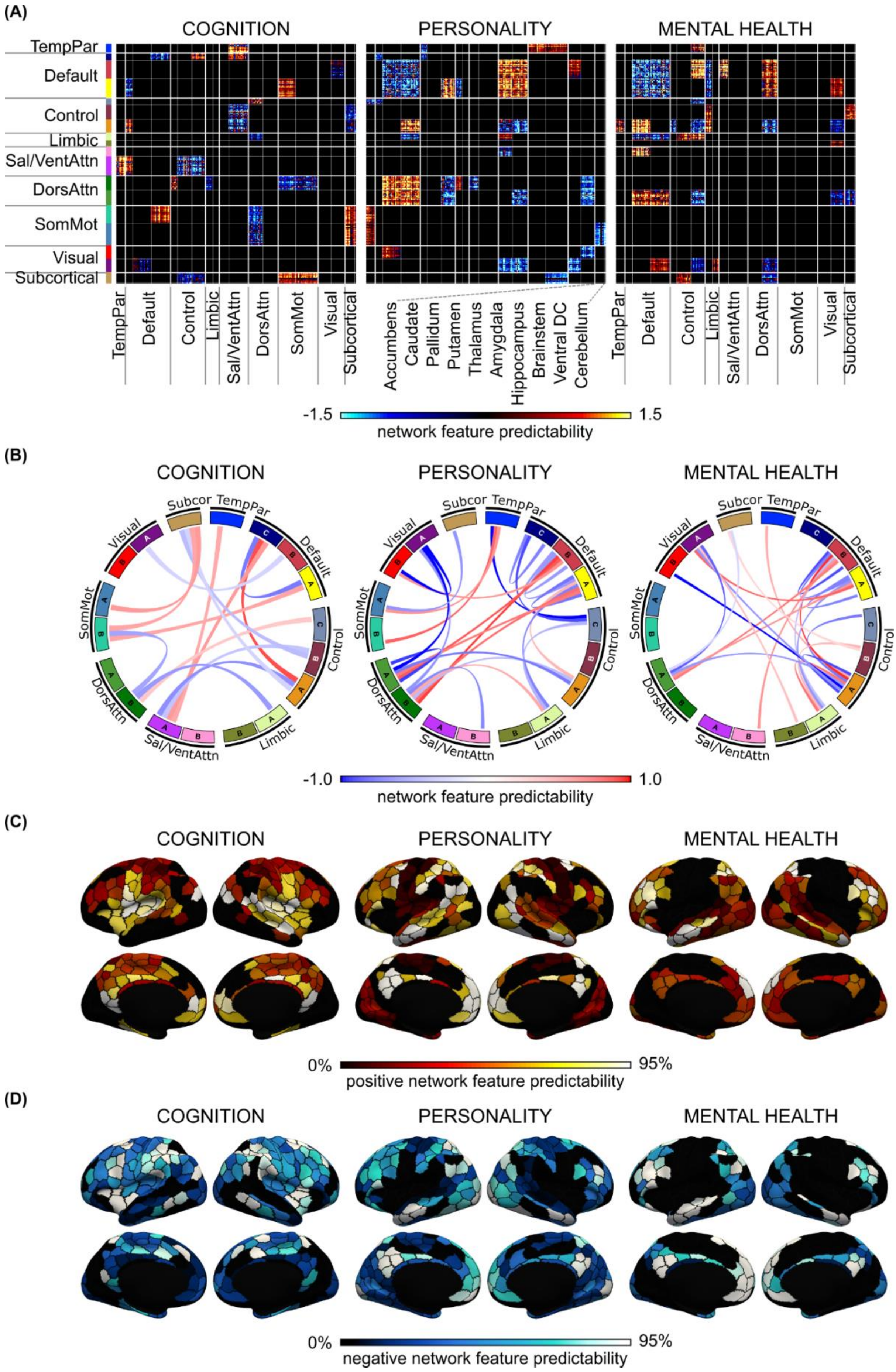
(A)
COGNITION
PERSONALITY
MENTAL HEALTH
TempPar
Default
Control
Limbic
Sal/VentAttn
DorsAttn
SomMot
Visual
Subcortical
Accumbens
Caudate
Pallidum
Putamen
Thalamus
Amygdala
Hippocampus
Brainstem
Ventral DC
Cerebellum
-1.5
1.5
network feature predictability
(B)
COGNITION
PERSONALITY
MENTAL HEALTH
Subcor
TempPar
Default
Control
Limbic
Sal/VentAttn
DorsAttn
SomMot
Visual
-1.0
1.0
network feature predictability
(C)
COGNITION
PERSONALITY
MENTAL HEALTH
0%
95%
positive network feature predictability
(D)
COGNITION
PERSONALITY
MENTAL HEALTH
0%
95%
negative network feature predictability

Supplementary Figure 22. Brain network features that support individual-level prediction of cognition, personality and mental health. This figure is the same as Figure 8 but using data processed with respiratory pseudo-motion filtering and more liberal quality control thresholds (N = 3744). (A) Predictive-feature matrices averaged across brain states, considering only within-network and between-network blocks that were significant across all four brain states (Rest, MID, SST, N-Back). (B) Predictive network connections obtained by averaging the matrices in panel (A) within each between-network and within-network block. (C) Positive predictive features obtained by summing positive predictive-feature values across the rows of panel (A). A higher value for a brain region indicates that stronger connectivity yielded a higher prediction for the behavioral measure. (D) Negative predictive features obtained by summing negative predictive-feature values across the rows of panel (A). A higher value for a brain region indicates that weaker connectivity yielded a greater prediction for the behavioral measure. In both panels C and D, the color of each parcel corresponds to the percentile of predictive-feature values among 400 parcels. For visualization, the values within each predictive-feature matrix in panel A were divided by their standard deviations across all entries in the predictive-feature matrix. Conclusions were highly similar using data from original processing pipeline (Figure 8).

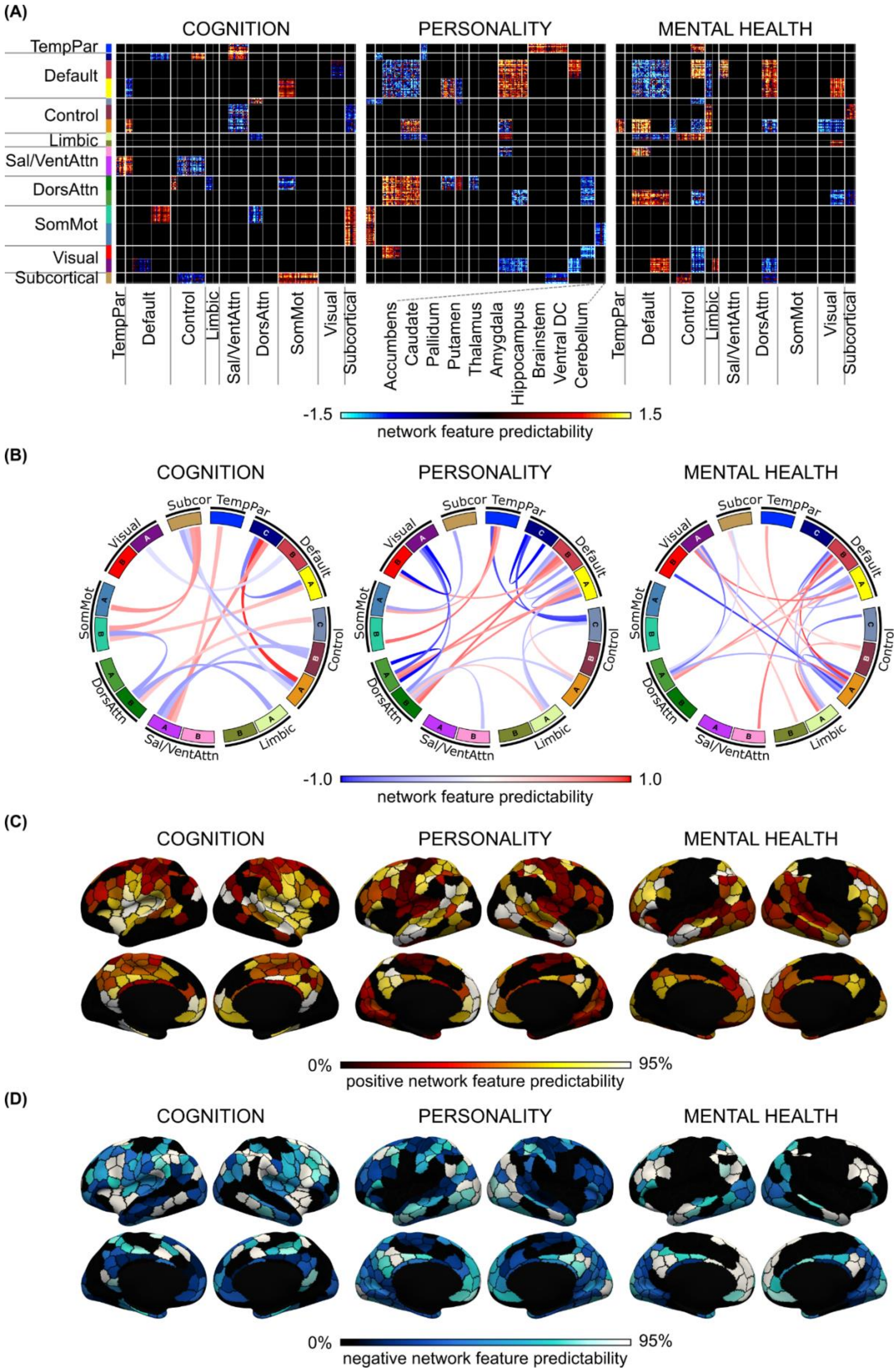

(A)
COGNITION
PERSONALITY
MENTAL HEALTH
TempPar
Default
Control
Limbic
Sal/VentAttn
DorsAttn
SomMot
Visual
Subcortical
Accumbens
Caudate
Pallidum
Putamen
Thalamus
Amygdala
Hippocampus
Brainstem
Ventral DC
Cerebellum
-1.5
1.5
network feature predictability
(B)
COGNITION
PERSONALITY
MENTAL HEALTH
Subcor
TempPar
Default
Control
Limbic
Sal/VentAttn
DorsAttn
SomMot
Visual
-1.0
1.0
network feature predictability
(C)
COGNITION
PERSONALITY
MENTAL HEALTH
0%
95%
positive network feature predictability
(D)
COGNITION
PERSONALITY
MENTAL HEALTH
0%
95%
negative network feature predictability

Supplementary Figure 23. Brain network features that support individual-level prediction of cognition, personality and mental health. This figure is the same as Figure 8 but using participants matched for age, sex, race, family income, and behavior with the overall population (Supplementary Table 9). (A) Predictive-feature matrices averaged across brain states, considering only within-network and between-network blocks that were significant across all four brain states (Rest, MID, SST, N-Back). (B) Predictive network connections obtained by averaging the matrices in panel (A) within each between-network and within-network block. (C) Positive predictive features obtained by summing positive predictive-feature values across the rows of panel (A). A higher value for a brain region indicates that stronger connectivity yielded a higher prediction for the behavioral measure. (D) Negative predictive features obtained by summing negative predictive-feature values across the rows of panel (A). A higher value for a brain region indicates that weaker connectivity yielded a greater prediction for the behavioral measure. In both panels C and D, the color of each parcel corresponds to the percentile of predictive-feature values among 400 parcels. For visualization, the values within each predictive-feature matrix in panel A were divided by their standard deviations across all entries in the predictive-feature matrix. Conclusions were highly similar using data from original analysis (Figure 8).

	REST	MID	N-Back	SST	Conjunction
Pass T1 recon-all qc	11075				
Not Phillips scanner	9640				
>= 2 Runs passed MRI QC	9217	8281	8141	8147	7495
Mean FD < 0.9 for both runs	8769	7271	6644	6908	5615
Having at least one minimally processed run released by ABCD	8451	6896	6287	6503	5014
No Missing behavior					4664
No Sibling					4187

Supplementary Figure 24. Number of participants if we used quality control (QC) criteria from Chaarani and colleagues (Chaarani et al., 2021) excluding task activation QC criteria (e.g., beta weights outlier detection). Because we excluded the task activation QC criteria in this table, the resulting sample size for each modality (e.g., N = 6503 for SST task) was larger than Chaarani's study. Yet, after conjunction across resting and task states, we were left with 4187 participants, which was only 11% more than our control analysis (N = 3744). Furthermore, we note that the QC criteria in this Figure excluded typical functional connectivity QC used in the literature.

Supplementary references

Acker, W. & Acker, C. Bexley Maudsley automated processing screening and Bexley Maudsley category sorting test manual (NFER-Nelson, 1982)

Hodes, R. J., Insel, T. R. & Landis, S. C., NIH Blueprint for Neuroscience Research. The NIH toolbox: setting a standard for biomedical research. *Neurology* **80**, S1 (2013).

Kawaguchi, K., Kaelbling, L. P. & Lozano-Pérez, T. Bayesian optimization with exponential convergence. *Adv. Neural Inf. Process. Syst.* **28**, 2809–2817 (2015).

Lynam, D. R. *Development of a short form of the UPPS-P Impulsive Behavior Scale*. Unpublished Technical Report (2013).

Pagliaccio, D. et al. Revising the BIS/BAS Scale to study development: Measurement invariance and normative effects of age and sex from childhood through adulthood. *Psychol. Assess.* **28**, 429–442 (2016).

Strauss, E., Sherman, E. M. S. & Spreen, O. *A Compendium of Neuropsychological Tests: Administration, Norms, and Commentary* (Oxford University Press, 2006).

Wechsler, D. Wechsler intelligence scale for children--Fifth Edition (WISC-V) (Pearson, 2014).
