# Supplementary material for: Shared and unique brain network features predict cognitive, personality, and mental health scores in the ABCD study
Source: Nat Commun. 2022 Apr 25;13:2217. doi: 10.1038/s41467-022-29766-8 (PMC9038754; doi:10.1038/s41467-022-29766-8)
Supplement: Supplementary file 3 — Reporting Summary [file 41467_2022_29766_MOESM3_ESM.pdf]

Corresponding author(s): B.T. Thomas Yeo

Last updated by author(s): Feb 3, 2022

Reporting Summary

Nature Research wishes to improve the reproducibility of the work that we publish. This form provides structure for consistency and transparency in reporting. For further information on Nature Research policies, see our Editorial Policies and the Editorial Policy Checklist.

Statistics

For all statistical analyses, confirm that the following items are present in the figure legend, table legend, main text, or Methods section.

n/a	Confirmed	
☐	☒	The exact sample size (*n*) for each experimental group/condition, given as a discrete number and unit of measurement
☐	☒	A statement on whether measurements were taken from distinct samples or whether the same sample was measured repeatedly
☐	☒	The statistical test(s) used AND whether they are one- or two-sided<br>*Only common tests should be described solely by name; describe more complex techniques in the Methods section.*
☐	☒	A description of all covariates tested
☐	☒	A description of any assumptions or corrections, such as tests of normality and adjustment for multiple comparisons
☐	☒	A full description of the statistical parameters including central tendency (e.g. means) or other basic estimates (e.g. regression coefficient) AND variation (e.g. standard deviation) or associated estimates of uncertainty (e.g. confidence intervals)
☐	☒	For null hypothesis testing, the test statistic (e.g. F, t, r) with confidence intervals, effect sizes, degrees of freedom and P value noted<br>*Give P values as exact values whenever suitable.*
☒	☐	For Bayesian analysis, information on the choice of priors and Markov chain Monte Carlo settings
☒	☐	For hierarchical and complex designs, identification of the appropriate level for tests and full reporting of outcomes
☐	☒	Estimates of effect sizes (e.g. Cohen's d, Pearson's r), indicating how they were calculated

Our web collection on statistics for biologists contains articles on many of the points above.

Software and code

Policy information about availability of computer code

Data collection	The data used came from a public dataset (Adolescent Brain Cognitive Development study). Data are collected via paper and pencil tests, iPad tasks, interview, and MRI scans. Full information on the data collection can be found at https://abcdstudy.org/
Data analysis	FreeSurfer (5.3.0); FSL (5.0.8); MATLAB (2018b); Python (3.6); Custom codes available in Zenodo under access code 5908961

For manuscripts utilizing custom algorithms or software that are central to the research but not yet described in published literature, software must be made available to editors and reviewers. We strongly encourage code deposition in a community repository (e.g. GitHub). See the Nature Research guidelines for submitting code & software for further information.

Data

Policy information about availability of data

All manuscripts must include a data availability statement. This statement should provide the following information, where applicable:

- Accession codes, unique identifiers, or web links for publicly available datasets
- A list of figures that have associated raw data
- A description of any restrictions on data availability

The ABCD data are publicly available via the NIMH Data Archive (NDA). Processed data from this study (including the predictive network features and FC matrices) have been uploaded to the NDA. Researchers with access to the ABCD data will be able to download the data: https://nda.nih.gov/study.html?id=824. Source data are provided with paper.

Field-specific reporting

Please select the one below that is the best fit for your research. If you are not sure, read the appropriate sections before making your selection.

☐ Life sciences ☒ Behavioural & social sciences ☐ Ecological, evolutionary & environmental sciences

For a reference copy of the document with all sections, see nature.com/documents/nr-reporting-summary-flat.pdf

Behavioural & social sciences study design

All studies must disclose on these points even when the disclosure is negative.

Study description	Quantitative cross-sectional study where we train and test predictive models to predict behavioral outcomes from neuroimaging data
Research sample	Male and female 9-10 year old children who were recruited from the Adolescent Brain Cognitive Development (ABCD) study, which is publicly available. We chose this sample because of its large sample size (n ~ 11,000) and because we were interested in predicting behavioral outcomes in children. After quality control, the main analysis utilized 1858 healthy unrelated children (55% female and 45% male).
Sampling strategy	11,875 ABCD participants were recruited from various communities in the United States and the participants will be followed for the next 10 years. The ABCD states they aimed for a sample that was large enough to accurately reflect the demographic variation of American adolescents.
Data collection	Data are collected via paper and pencil tests, iPad tasks, interview, and MRI scans. Full information on the data collection can be found at https://abcdstudy.org/
Timing	The data collection for this public dataset is still on-going. The baseline data used in the current paper were collected between September 2016 and October 2018.
Data exclusions	We excluded ~9k participants from our study because of missing data (e.g. many participants do not have all the desired measures) or poor quality data. More details are found in Figure 1A of the paper.
Non-participation	This information is not available to us since we were not involved in the data collection.
Randomization	We did not allocate participants into different experimental groups because they all underwent the same experimental conditions.

Reporting for specific materials, systems and methods

We require information from authors about some types of materials, experimental systems and methods used in many studies. Here, indicate whether each material, system or method listed is relevant to your study. If you are not sure if a list item applies to your research, read the appropriate section before selecting a response.

Materials & experimental systems

n/a	Involved in the study	
☒	☐	Antibodies
☒	☐	Eukaryotic cell lines
☒	☐	Palaeontology and archaeology
☒	☐	Animals and other organisms
☐	☒	Human research participants
☒	☐	Clinical data
☒	☐	Dual use research of concern

Methods

n/a	Involved in the study	
☒	☐	ChIP-seq
☒	☐	Flow cytometry
☐	☒	MRI-based neuroimaging

Human research participants

Policy information about studies involving human research participants

Population characteristics	See above
Recruitment	Participants are recruited through the school systems and the school selection are informed by demographic characteristics to minimize selection bias. Participants are reimbursed and the reimbursement rates varies across sites based on the costs of living. Typical compensation includes $200 for the parent / guardian and $100 worth of gifts for the child. See Garavan 2018 for more details.
Ethics oversight	The ABCD study was approved by the Institutional Review Board at University of California, San Diego. Parents or guardians provided written consent while the child provided written assent.

Note that full information on the approval of the study protocol must also be provided in the manuscript.

Magnetic resonance imaging

Experimental design

Design type	functional imaging (a combination of block and event-related task and resting-state)
Design specifications	Each participant underwent 4 runs of resting-state and 6 runs of tasks. The timing of the runs is variable depending on the task.
Behavioral performance measures	While performance measures (e.g. correct button press, response times) were collected during the task imaging sessions, we did not use these outcomes in our current study. We used behavioral measures that were collected outside of the scanner.

Acquisition

Imaging type(s)	functional
Field strength	3T
Sequence & imaging parameters	Multiple scanners of different makes were used, so the sequences were variable, but each fMRI scan was in 2.4 mm isotropic resolution with a TR of 800 ms. Please see Casey et al 2018 Developmental Cognitive Neuroscience for more details.
Area of acquisition	Whole brain scan

Diffusion MRI ☐ Used ☒ Not used

Preprocessing

Preprocessing software	FreeSurfer 5.3.0; FSL 5.0.8; Our preprocessing code can be found here: https://github.com/ThomasYeoLab/CBIG/tree/master/stable_projects/preprocessing/CBIG_fMRI_Preproc2016
Normalization	We aligned the fMRI data to the T1 images using boundary-based registration (Greve and Fischl 2009, NeuroImage) with FsFast. Cortical surfaces were extracted from the T1 images and aligned to FreeSurfer fsaverage space using FreeSurfer.
Normalization template	FreeSurfer fsaverage6 surface space
Noise and artifact removal	We regressed out the global signal, six motion correction parameters, averaged ventricular signal, averaged white matter signal, and their temporal derivatives (18 regressors in total)
Volume censoring	Volumes with a frame displacement > 0.3 mm (Jenkinson et al. 2002 NeuroImage) or voxel-wise differentiated signal variance (DVARS) > 50 (Power et al. 2012 NeuroImage), along with one volume before and two volumes after, were marked as outliers and subsequently censored.

Statistical modeling & inference

Model type and settings	predictive
Effect(s) tested	We tested whether we could predict behavioral outcomes from functional connectivity derived from task and resting-state fMRI.

Specify type of analysis: ☒ Whole brain ☐ ROI-based ☐ Both

Statistic type for inference (See Eklund et al. 2016)	We use functional connectivity measures in our study, so we were not interested in clusters from task-based activations.
Correction	False Discovery Rate

Models & analysis

n/a	Involved in the study	
☐	☒	Functional and/or effective connectivity
☒	☐	Graph analysis
☐	☒	Multivariate modeling or predictive analysis

Functional and/or effective connectivity	Pearson correlation
Multivariate modeling and predictive analysis	We used kernel ridge regression and evaluated the performance of our models with two metrics: correlation

Multivariate modeling and predictive analysis

between predicted and observed values, and coefficient of determination. For control analysis, we also utilized linear ridge regression.
